# A computational approach for inferring the cell wall properties that govern guard cell dynamics

**DOI:** 10.1111/tpj.13640

**Published:** 2017-08-23

**Authors:** Hugh C. Woolfenden, Gildas Bourdais, Michaela Kopischke, Eva Miedes, Antonio Molina, Silke Robatzek, Richard J. Morris

**Affiliations:** ^1^ Computational and Systems Biology John Innes Centre Norwich Research Park Norwich NR4 7UH UK; ^2^ The Sainsbury Laboratory Norwich Research Park Norwich NR4 7UH UK; ^3^ Centro de Biotecnología y Genómica de Plantas (CBGP) Universidad Politécnica de Madrid (UPM) Instituto Nacional de Investigación y Tecnología Agraria y Alimentaria (INIA) Campus de Montegancedo UPM 28223 Pozuelo de Alarcón Madrid Spain; ^4^ Departamento de Biotecnología‐Biología Vegetal Escuela Técnica Superior de Ingeniería Agrónomica Alimentaria y de Biosistemas, UPM 28040 Madrid Spain

**Keywords:** guard cells, cell wall, computational modelling, biomechanics, stomata, *Vicia faba*, *Arabidopsis thaliana*

## Abstract

Guard cells dynamically adjust their shape in order to regulate photosynthetic gas exchange, respiration rates and defend against pathogen entry. Cell shape changes are determined by the interplay of cell wall material properties and turgor pressure. To investigate this relationship between turgor pressure, cell wall properties and cell shape, we focused on kidney‐shaped stomata and developed a biomechanical model of a guard cell pair. Treating the cell wall as a composite of the pectin‐rich cell wall matrix embedded with cellulose microfibrils, we show that strong, circumferentially oriented fibres are critical for opening. We find that the opening dynamics are dictated by the mechanical stress response of the cell wall matrix, and as the turgor rises, the pectinaceous matrix stiffens. We validate these predictions with stomatal opening experiments in selected Arabidopsis cell wall mutants. Thus, using a computational framework that combines a 3D biomechanical model with parameter optimization, we demonstrate how to exploit subtle shape changes to infer cell wall material properties. Our findings reveal that proper stomatal dynamics are built on two key properties of the cell wall, namely anisotropy in the form of hoop reinforcement and strain stiffening.

## Significance Statement

We describe a computational approach for investigating guard cell dynamics, focusing on the link between turgor pressure, geometry and the material properties of the cell walls. Our results show that radially‐arranged fibers are critical for stomatal opening and that the cell wall matrix stiffens as turgor rises.

## Introduction

Stomata are microscopic pores formed by a pair of guard cells in the plant epidermis, through which plants control gas exchange with their environment. Guard cells regulate the pore size in response to abiotic and biotic stimuli by contracting [e.g. in response to darkness, high CO_2_ concentration, drought, abscisic acid (ABA) and microbe‐associated molecular patterns] or expanding (e.g. in response to blue and red light, low CO_2_ concentration, high humidity, pathogen‐secreted effectors) (Webb *et al*., 2001; Kim *et al*., 2010; Kollist *et al*., 2014; McLachlan *et al*., 2014). Guard cells regulate the opening and closing of the stomata as a function of pressure, which is governed by the osmotic potential that in turn can be changed by altering intracellular ion concentrations via membrane channels, pumps and transporters (Kollist *et al*., 2014).

Turgor pressure in fully open *Vicia faba* guard cells is around 50 atmospheres (e.g. Franks *et al*., 1998; Franks, 2003). To withstand such pressure, plant cells have a strong extracellular structure, the cell wall, consisting of a mesh of cross‐linked cellulose microfibrils (CMFs) embedded in a hemicellulose and pectin matrix (Somerville *et al*., 2004; Cosgrove, 2016). To model the biomechanics of stomatal opening and closing we need to know their cell wall material properties. Ranges for the elastic moduli of the cell wall components, e.g. cellulose, lignin and hemicellulose, are known, and the modulus for the whole tissue lies in the range spanned by the moduli, with the value dependent on the relative abundance of each component (Gibson, 2012). A variety of experimental techniques have been used to quantify elastic moduli in plants (reviewed by Vogler *et al*., 2015). For example, tension devices have been used to test cell wall sheets of *Chara corallina* (Toole *et al*., 2001), plant cell wall analogues (Chanliaud *et al*., 2002), Arabidopsis hypocotyls (Ryden *et al*., 2003) and *Prunus avium* (sweet cherry) fruit skin (Bargel *et al*., 2004). More recently, elastic moduli estimates have been obtained for Arabidopsis leaves (Hayot *et al*., 2012; Forouzesh *et al*., 2013) and *Allium* (onion) epidermal peels (Beauzamy *et al*., 2015) by nanoindentation techniques, at the subcellular scale using microelectromechanical system devices (Zamil *et al*., 2013), and for growing *Camellia* pollen tubes using microfluidic devices (Nezhad *et al*., 2013a,b). Thus, although there is considerable variation (Cosgrove, 2016), there is a wealth of information about cell wall moduli and in particular for the epidermis; however, the cell wall composition in guard cells differs significantly to that of epidermal cells (Jones *et al*., 2004; Amsbury *et al*., 2016), which might suggest different behaviour, yet it is not clear how changes in the cell wall components alter the overall cell dynamics. To understand how guard cells function we need to understand how the cell wall material properties translate turgor into a reversible biomechanical response (Weber *et al*., 2015).

The early work on stomatal modelling by Cooke *et al*. (1976) treated a stoma as a thin‐shelled torus. As guard cells return to their initial shape when turgor pressure is reduced, i.e. in terms of their geometry they reversibly open and close, Cooke *et al*. (1976) approximated the guard cell walls as an elastic material. Within this framework, an important choice is the type of elastic model to use, which can be linear or nonlinear. The fundamental difference between the theory of linear and nonlinear elasticity is the size of deformation to which it can be applied. Linear elasticity is a suitable approximation only for small deformations (on the order of a few percent). The model of Cooke *et al*. (1976) used linear elasticity but stated that nonlinear elasticity would be a logical progression. Their model gave rise to several notable conclusions, including that variations in cell wall thicknesses were not required for stomatal opening, contrary to the thinking of the time (Niklas, 1992). A further conclusion was that radial cell wall stiffening, provided by CMFs, was not essential for proper stomatal function (when a pore opens as guard cell turgor pressure increases), provided the cell wall was sufficiently thick. Radially aligned CMFs have been observed in guard cell walls across species (Palevitz and Hepler, 1976; Fujita and Wasteneys, 2013). When aligned CMFs are incorporated into the cell wall, the stiffness becomes direction dependent, or anisotropic (Aylor *et al*., 1973; Wu and Sharpe, 1979). When stomata open, these radially aligned CMFs would be expected to act against any widening of guard cell cross section, and instead, direct the increase in turgor pressure into a longitudinal lengthening of the guard cell. This behaviour has been observed by Meckel *et al*. (2007), who showed that *V. faba* guard cells lengthened longitudinally as the stoma opened, with only a slight increase in guard cell cross section. They found that guard cells increase in volume by 25% or more during stomatal opening (Franks *et al*., 2001; Meckel *et al*., 2007). As linear elastic theory is inappropriate when deformations are large, nonlinear elasticity is required to account for the observed shape changes.

Here we use a theoretical approach and develop a minimal biomechanical model that quantitatively captures the opening dynamics of kidney‐shaped stomata. Building on previous work (e.g. Aylor *et al*., 1973; DeMichele and Sharpe, 1973; Cooke *et al*., 1976; Wu and Sharpe, 1979), we extend these investigations to allow for spatial variations in cell wall thicknesses, a nonlinear anisotropic mechanical model, and computation of the mechanical stress and strain throughout the cell wall. Furthermore, we put forward a framework for the inference of biomechanical properties of the cellulose microfibrils and the pectin‐rich cell wall matrix, and how they affect stomatal function (Rui and Anderson, 2016). Our simulations confirm that microfibrils are critical for stomatal function, and we predict that the microfibrils must be strong in order to impart sufficient hoop rigidity to the guard cell wall, and that the cell wall matrix strain‐stiffens during opening. By applying the model to Arabidopsis we confirm that stomata of wild‐type plants and selected mutants require strong microfibrils, and that a mutant with an enhanced opening phenotype, and enriched in a pectin (previously implicated in altered stomatal dynamics), had a less stiff cell wall matrix compared with the wild type. The model can therefore not only answer questions and make predictions regarding isolated stomata but can also complement experimental observations, thereby leading to an improved understanding of guard cell dynamics.

## Results

### Anisotropic guard cell walls are critical for stomatal function

**Figure 1:**
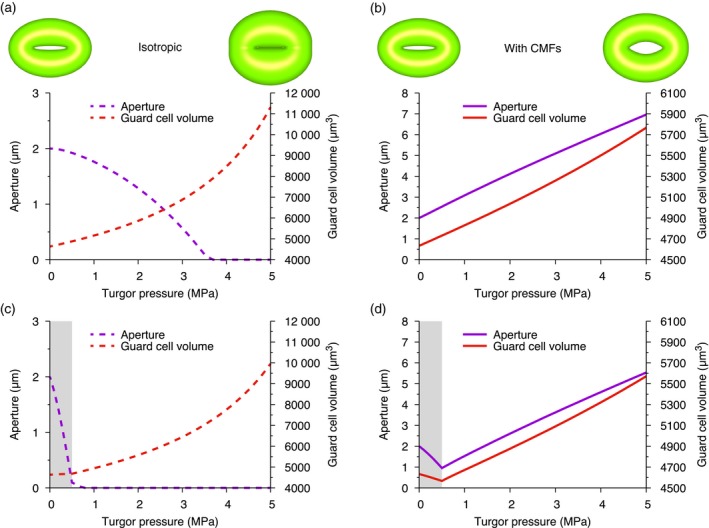
Circumferentially oriented cellulose fibres are critical for proper stomatal function. The turgor pressure is increased from 0 to 5 MPa in two geometrically identical stomata. (a) When the cell wall is isotropic the guard cells swell and the stoma closes its pore. (b) The aperture and guard cell volume increase for a stoma with circumferential cellulose microfibrils (CMFs) in the guard cell walls. (c) Repeat of (a) with epidermal pressure applied to the dorsal wall. (d) Repeat of (b) with epidermal pressure applied. (c, d) Grey shaded area indicates the range of epidermal pressure. The stomatal dimensions are given in Table 1 and the cell wall parameters are either the ‘Isotropic’ or ‘With CMFs’ values given in Table 2. Stomata images show the initial and final shapes, and are shaded to provide perspective.

In order to understand the impact of the cell wall properties during opening and closing, we constructed a three‐dimensional biomechanical model of a guard cell pair. To account for the large deformations observed during opening (Franks *et al*., 2001; Meckel *et al*., 2007), we employed nonlinear elasticity theory. The model is shown in Figure S1 and is described in detail in Experimental procedures. We used the stomatal measurements for *V. faba* provided by Spence *et al*. (1986), and listed in Table 1, to generate the initial stomatal configuration shown in Figure 1(a). The model is initialized so that the stoma length is 45 μm, the guard cell width is 15 μm, and the Young's modulus and Poisson's ratio are approximately 108 MPa and 0.5, respectively. First, to validate the findings of Cooke *et al*. (1976), we set the cell wall to be isotropic, i.e. the elastic modulus is independent of direction. To simulate what happens when guard cells take up water, we applied turgor pressure to the inner cell wall surfaces (details regarding the pressure range are presented in the Experimental procedures). The resulting pressurized, deformed configuration, together with quantification of the guard cell volume and pore aperture, which both change in a nonlinear fashion, are shown in Figure 1(a). Importantly, we find that for an isotropic cell wall the pore does not open with increasing turgor but actually closes. This result seems to be in conflict with the conclusion of Cooke *et al*. (1976) that an isotropic cell wall can produce a functional stoma, provided the cell wall was thick enough. We therefore investigated the impact of cell wall thickness. We doubled the cell wall thickness and also investigated doubling the thickness of the ventral wall whilst leaving the dorsal and periclinal wall thicknesses unchanged – the guard cell cross sections are shown for these models in Figure S2. The latter case mimics the observation that the ventral wall tends to be thicker than the dorsal wall in some stomata species (Franks and Farquhar, 2007a). We find that neither case qualitatively changes the behaviour of the stoma, i.e. the pore closes as turgor increases (Figure S2). As expected, we find that as the thickness increases, a higher turgor pressure is required to achieve the same deformation. A qualitatively similar result occurs if the stiffness of the cell wall matrix is increased without changing the initial cell wall thickness (Figure S2). Therefore, increasing the cell wall thickness or raising the cell wall stiffness produces the same effect, and neither explain the difference between our results and those of Cooke *et al*. (1976).

**Table 1:** Measurements from experiments and the model for *Vicia faba* stomata

		Closed stoma		Open stoma		
	Units	Observed	Model	Observed	Model #1	Model #2
Stoma length	μm	45.0	45.0	47.0	50.5 (+7.5%)	51.6 (+9.8%)
Pore length	μm	17.0	17.0	22.0	22.2 (+0.9%)	22.4 (+2.0%)
Aperture	μm	2.0	2.0	12.5	12.2 (−2.5%)	12.1 (−3.1%)
Guard cell width	μm	15.0	15.0	15.0	15.3 (+2.2%)	15.4 (+2.7%)

The observed measurements are for the large, and most common, *V. faba* stoma in Spence *et al*. (1986). The values for the closed stoma are used to construct the undeformed stomatal geometry in the model. The difference between the ‘Model #1’ and the ‘Model #2’ results arises from different cell wall matrix models, where the latter model incorporates stiffening. For the closed stoma, the dorsal and ventral wall thicknesses were set to 1 μm, which is in the range observed by Li *et al*. (2010). The tip wall thickness was set to 0.3 μm, as observed by Meckel *et al*. (2007).

To investigate this discrepancy in behaviour we initialized the guard cell geometry so that the guard cells bulge into the plane of the leaf, i.e. the aspect ratio (AR) of the guard cell cross section is less than one (AR = guard cell depth/guard cell width). Increasing the turgor for this configuration initially leads to a slight increase in aperture as the guard cells lose their eccentric cross section, but as the turgor rises further the stoma closes (Figure S3). The former situation corresponds to the behaviour observed by Cooke *et al*. (1976). Had they increased the turgor pressure to what we now know is the correct range, their guard cell model would also have likely closed rather than opened. Unsurprisingly, it's the interplay between shape, pressure and material that determines morphological changes. For example, Hofhuis *et al*. (2016) showed that the mechanism underlying explosive seed dispersal relies not only on geometry and pressure, but also on the material properties of the epidermal wall. The conclusion of Cooke *et al*. (1976) relating to the proper functioning of stomata with isotropic cell walls is thus shown to result from their choice of initial geometry and limited pressure range (Figure S3).

As we failed to find parameters for an isotropic material with which guard cells can function correctly, we extended the model to allow for anisotropic behaviour (Dumais *et al*., 2006). Anisotropy describes the direction‐dependent response of a material to an applied force, behaviour that is imparted into guard cell walls by circumferentially‐oriented CMFs. We include CMFs in the model by adding circumferential fibres to the cell wall (Figure S1b). The cell wall, therefore, becomes a composite of two components in our model: an isotropic cell wall matrix and fibres embedded in this matrix that provide anisotropy parameterized by the fibre strength and direction. The fibre parameters are representative of the fibre network as a whole, i.e. CMFs, hemicellulose, linking and unlinking rates, etc., rather than the modulus of an isolated cellulose fibril, which is ≈100 GPa (Cheng and Wang, 2008). This anisotropic cell wall model is similar to the recent investigation of Weber *et al*. (2015), who developed a continuum mechanics‐based approach to model a cylindrical *Nicotiana benthamiana* (tobacco) cell as a thin shell, and the approach of Bozorg *et al*. (2014), who idealized the cell wall in the growing shoot apical meristem as a mesh of triangular biquadratic springs using thin shells.

To ensure that the guard cell walls are anisotropic we chose the fibre modulus to be high (≈10×) relative to the initial Young's modulus of the cell wall matrix. We then increased the turgor pressure in the guard cells to deform the stoma from its initial configuration and quantified the change in aperture and guard cell volume (Figure 1b). With CMFs providing hoop rigidity, the model captures the stoma opening its pore. A further consequence of the hoop rigidity is a restriction on the increase in guard cell volume to ≈25%, which is in agreement with experimental data (Franks *et al*., 2001; Meckel *et al*., 2007). To investigate the impact of pressurized subsidiary cells on guard cell behaviour, we studied the opening dynamics with an external pressure applied to the dorsal walls. When we don't include CMFs in the model, the presence of epidermal pressure has a significant effect on the guard cell dynamics. With no CMFs and no epidermal pressure, the stoma closes at a guard cell turgor pressure of ≈4 MPa (Figure 1a), whereas with epidermal pressure the stoma closes at ≈0.5 MPa (Figure 1c). When we include CMFs in the model, however, the stoma closes slightly as the epidermal pressure rises to its maximum value (0.5 MPa), after which point the stoma opens normally (Figure 1d). As the model stoma opens, the impact of epidermal pressure on the aperture diminishes, which is in line with experimental observations (Franks *et al*., 1998). In accordance with our previous results, thickening the cell wall or increasing the stiffness of the matrix reduces the response for a given pressure (Figure S2). Furthermore, anisotropic guard cells open under pressure, irrespective of the initial aspect ratio of the guard cell cross section (Figure S3). Anisotropy in the walls of guard cells is, therefore, a fundamental requirement of a functional stoma.

### Strong cellulose microfibrils are required for stomatal opening

In order to estimate the elastic moduli that can reproduce stomatal dynamics, we introduced a measure for the ‘distance’ of the model‐derived geometry from the experimentally observed geometry, and used standard optimization techniques to minimize this difference by changing the elastic moduli of the cell wall. Details of this process can be found in the Experimental procedures. The result of the optimization is a set of parameters for the cell wall matrix and the CMFs that, as pressure rises, best reproduce the experimentally observed open stoma shapes.

We initialized the optimization procedure using the closed *V. faba* stoma described by Spence *et al*. (1986), and sought elastic moduli that produced the best match to the open stoma when the turgor pressure was raised. The dimensions for the open and closed stoma are summarized in Table 1. The cost function includes the stoma and pore length together with the aperture and the guard cell width. As the material model decouples the cell wall matrix from the CMFs, the optimization procedure finds elastic moduli specific to the cell wall matrix and to the CMFs (Table 2). The small‐strain shear modulus, *G*_0_, of the cell wall matrix is found to be 18 MPa, corresponding to a Young's modulus of 54 MPa. This estimate can be compared with known materials, e.g. it is greater than the shear modulus of rubber (0.3 MPa) but smaller than the modulus of polyethylene (120 MPa). The shear modulus, *G*, measures the ability of a substance to resist a shearing force, and *G*_0_ is the value of *G* at the onset of deformation: for a nonlinear material *G* typically deviates from *G*_0_ during the course of deformation. The value of the inferred fibre modulus is ≈1.2 GPa, which is several orders of magnitude larger than *G*_0_, highlighting the difference in strengths of the cell wall matrix and the CMFs, which is consistent with a recent study (Sampathkumar *et al*., 2014). These values are estimates within the framework of our model, which includes approximations such as simplified geometry, having only one type of fibre with idealized alignment and no cross‐linking, and material incompressibilty. Within this framework, the high strength of the circumferential CMFs, relative to the matrix, is crucial for restricting radial expansion of the guard cells, consistent with experimental data that show guard cell widths remain constant during opening (Spence *et al*., 1986; Rui and Anderson, 2016). This approach allows us to accurately recapitulate the open stoma, with the aperture, guard cell width and pore length all within 2.5% of the observed values, and the stoma length about 7.5% longer than for the observed stoma. The analysis of the isotropic case and inferred parameters for the anisotropic case show that strong, circumferential CMFs are required in the guard cell walls in order for the stoma to function properly, and to match experimental observations.

**Table 2:** Cell wall parameters for the non‐stiffening cell wall matrix

	Cell wall matrix			Fibres
	*C*_1_ (MPa)	*C*_2_ (MPa)	*G*_0_ (MPa)	*C*_5_ (MPa)
Isotropic	9.00	9.00	36.0	–
With CMFs	9.00	9.00	36.0	1000
Inferred #1	4.45	4.48	17.9	1170

The parameters *C*_1_ and *C*_2_ can be viewed as fitting parameters for the cell wall matrix model, whereas *G*_0_ is the small‐strain shear modulus and *C*_5_ is the modulus of the cellulose microfibrils (CMFs; details in Experimental procedures). The values for the ‘Isotropic’ and ‘With CMFs’ sets are prescribed in the model. The ‘Inferred #1’ values are the results of the parameter optimization for the stomatal geometry in Table 1 using the non‐stiffening cell wall matrix model.

**Figure 2:**
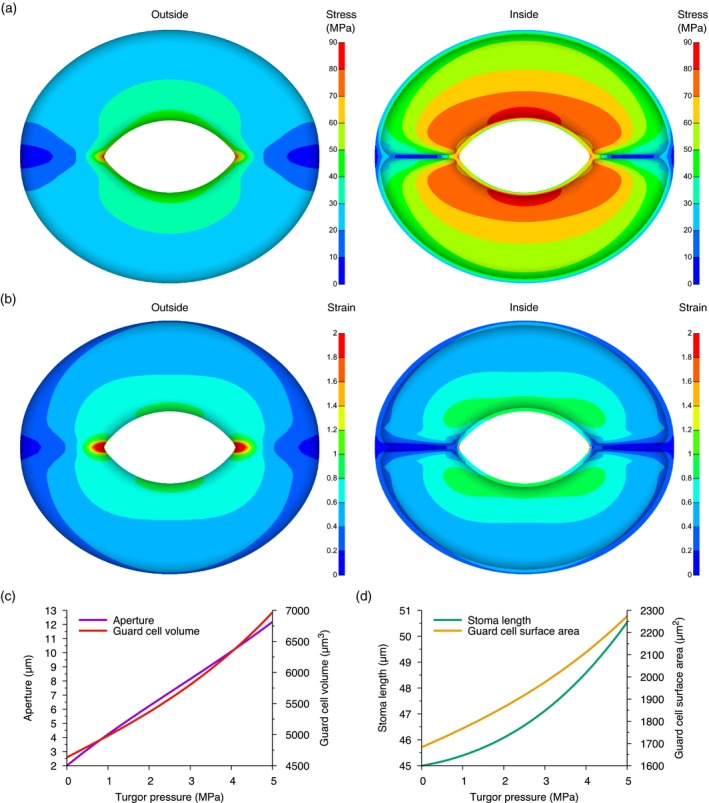
Stomatal opening induces stress and strain hot spots. (a) Distribution of the (effective) stress on the outside (left panel) and the inside (right panel) of the open stoma. (b) Distribution of the (effective Lagrange) strain on the outside (left panel) and the inside (right panel) of the stoma, with the strain limited to 2. (c) The increase in aperture (purple line) and guard cell volume (red line) as the turgor pressure increases from 0 to 5 MPa. (d) The increase in stoma length (green line) and inner surface area of a guard cell (yellow line) as pressure increases. The stomatal dimensions are given in Table 1 and the cell wall parameters are the ‘Inferred #1’ values in Table 2.

The open stomata geometry can be seen in Figure 2, together with a contour map of the mechanical stress and strain throughout the stoma; the inner and outer surfaces of the guard cells are shown. As can be seen, the ventral wall, particularly on the inside and where the tips of the guard cells join, experiences the greatest stress (Figure 2a). The strain distribution paints a similar picture (Figure 2b): strain ‘hot spots’ occur in the ventral wall where the tips join, and the strain is higher in the ventral wall than in the dorsal wall. These maps show the effective stress and strain, which summarize the tensors as scalar values. Vectorial views of the stress and strain tensors are shown in Figure S4 using the first principal stress and strain of the tensors. These clearly show that stress is directed circumferentially with the CMFs and the strain is oriented longitudinally, indicating the direction of maximum stretch. The overall surface area increases by 30% during opening (Figure 2d), and on the ventral wall this rises to 50% in places. The points where the tips meet the ventral walls are therefore important locations in stomatal opening dynamics, as they are hot spots of stress and strain. The high stress and strain at the tips indicate that the ventral wall may yield in this region and separate in order to form more tapered ends to the pore, deviating from the simple geometry that we chose. By analysing the stress–strain curve we estimated the Young's modulus at the guard cell mid‐point and at the end of the pore to be ≈59.9 and ≈11.2 MPa, respectively. The precise locations of the points at which these calculations were made are shown as point (a) and point (b) in Figure S1.

This 30% increase in surface area as the stoma opens is well above the reported values for biomembranes that stretch elastically by only 3–5% (Wolfe *et al*., 1986; Morris and Homann, 2001; Gao *et al*., 2005). For the plasma membrane, vigorous endo‐ and exocytosis in the regions towards the poles has been proposed (Meckel *et al*., 2004; Gall *et al*., 2010) as a mechanism that facilitates the change in surface area, whereas others propose that plasma membrane excretion and folding occurs during closure in addition to endo‐ and exocytosis (Li *et al*., 2010). The regions of high strain on the inside of the guard cells could be interpreted as mechanical cues as to where reinforcement is most required in order to maintain the integrity of the plasma membrane during stomatal opening. If stress and/or strain are being sensed by the cell wall (Heisler *et al*., 2010), then it is plausible that these hot spots might be a focus for cell wall strengthening. The 30% increase in surface area assumes that the cell wall is initially smooth, whereas if the surface were slightly rugose then the change in surface area required during stomatal opening would decrease. Whatever the underlying process, the model locates and quantifies stress and strain hot spots in guard cells as the stoma opens, highlighting the regions where folding of the plasma membrane and/or membrane recycling are most required to enable guard cells to achieve the observed change in surface area.

### The guard cell wall matrix strain‐stiffens during stomatal opening

Pressure‐probe experiments have shown that the aperture depends nonlinearly on turgor pressure, with the aperture least sensitive to a change in pressure in the highest part of the pressure range (Franks *et al*., 1998; Franks and Farquhar, 2007a). The nonlinear behaviour could be achieved by a dynamically changing cell wall thickness or by stress inducing structural rearrangements in the cell wall, and thus leading to changes in the material properties. Without further data we can't uncouple these effects and we therefore focus on the phenomological cell wall response to pressure, rather than the underlying details. As the guard cell volumetric elastic modulus (similar in definition to the bulk modulus; for a detailed description, see Cosgrove, 1988) has been observed to increase during stomatal opening (Franks *et al*., 2001), we asked whether strain‐stiffening of the cell wall might explain the nonlinear dependence of aperture on pressure. Strain‐stiffening has been discussed in relation to the stiffening of isolated, but growing, cell walls (Cosgrove, 1993), and has been shown to occur in the growing shoot tip (Kierzkowski *et al*., 2012). Within our model, strain‐stiffening could be achieved either as a property of the cell wall matrix or as a property of the CMFs.

To check whether fibre stiffening might lead to the nonlinear pressure–aperture profile, we performed simulations with strain‐stiffening fibres and the same cell wall matrix as before. This demonstrated that fibre stiffening mainly affected the guard cell width, whereby fibres that were permitted to stiffen as their embedding matrix stretched led to an increase in guard cell width, and did not reproduce the observed nonlinear opening behaviour. As we treat the cell wall as incompressible, the high stiffness in the circumferential direction could be transferred to the longtitudinal direction. Therefore, to further check whether strain‐stiffening fibres reproduce the observed nonlinear opening behaviour, we included the fibre strain‐stiffening parameter, λ_m_, in the material parameter optimization. This procedure resulted in fibres that did not strain‐stiffen, suggesting that strain‐stiffening CMFs do not give rise to the nonlinear opening behaviour.

**Figure 3:**
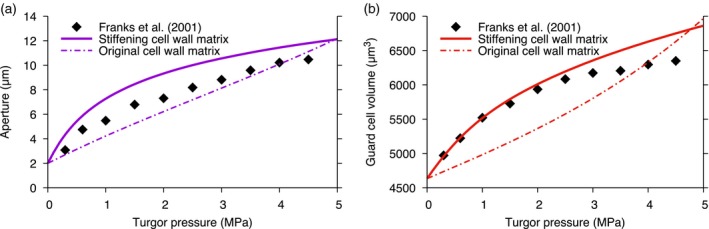
A strain‐stiffening cell wall matrix recapitulates nonlinear aperture and volume profiles. Change in the aperture (a) and the guard cell volume (b) as the turgor pressure increases from 0 to 5 MPa for the original cell wall matrix model (dash‐dot line) and the stiffening cell wall matrix (solid line). Results for a *Vicia faba* stoma from Franks *et al*. (2001) are shown for comparison (black diamonds). The stomatal dimensions used by the model are given in Table 1. The cell wall parameters are the ‘Inferred #1’ values in Table 2 for the original cell wall matrix model and the ‘Inferred #2’ values in Table 3 for the stiffening matrix model.

To evaluate whether the observed pressure–aperture profiles could be explained by strain‐stiffening of the cell wall matrix, we modified the mathematical relationship that links stress and strain in the matrix (details are presented in the Experimental procedures). With this model, we verified that the stoma functions correctly only when CMFs are present in the cell wall (Figures S5, S6), consistent with our previous inferences. This confirmed that strain‐stiffening does not replace the requirement for anisotropy in the cell wall. Then, we inferred the elastic moduli that produce the best match to the open stoma (model shown in Figure S6; Table 3). The variation of the aperture with respect to the pressure is shown in Figure 3 for both the previous and the updated cell wall matrix models. The almost linear pressure–aperture relationship of the original cell wall matrix model changes to a nonlinear profile for the stiffening cell wall matrix model, and the aperture becomes most sensitive to pressure changes in the lowest part of the pressure range, and is least sensitive at high pressures. This is precisely the relationship between aperture and pressure that is observed in pressure probe experiments when the stoma is surrounded by ruptured epidermal cells (Franks *et al*., 1998, 2001). Consistent with experiments on isolated pectin‐based gels that revealed strain‐stiffening behaviour as force is applied (see Braybrook *et al*., 2012, and references therein), our simulations suggest that strain‐stiffening of the cell wall matrix is possible, and provides an explanation for the nonlinear stomatal opening behaviour.

**Table 3:** Cell wall parameters for the stiffening cell wall matrix

	Cell wall matrix			Fibres
	*C*_1_ (MPa)	*C*_2_ (–)	*G*_0_ (MPa)	*C*_5_ (MPa)
Inferred #2	2.76	1.61	4.4	2320
Inferred Col‐0	1.55	21.28	33.0	601
Inferred *irx8*	0.98	48.88	48.1	714
Inferred *pmr5*	1.52	20.05	30.6	601
Inferred *pmr6*	0.99	20.00	19.7	705

The parameters *C*_1_ and *C*_2_ (dimensionless) can be viewed as fitting parameters for the cell wall matrix model, whereas *G*_0_ is the small‐strain shear modulus and *C*_5_ is the modulus of the cellulose microfibrils (CMFs; details in Experimental procedures). The ‘Inferred #2’ values are the result of the parameter optimization for *Vicia faba* for the stomatal geometry in Table 1, using the stiffening cell wall matrix model. The values for the *Arabidopsis thaliana* genotypes, Col‐0, *irx8*,* pmr5* and *pmr6*, were inferred using the mean geometry of the experimental data (shown in Table 4) and the material parameter optimization procedure.

Strain‐stiffening results in a small‐strain shear modulus, *G*_0_, of the matrix of 4.4 MPa, which corresponds to a Young's modulus (*E*) of 13.2 MPa. This value of *G*_0_ is about 4× lower than the value for the original matrix model (Tables 2, 3). As the change in aperture now saturates at high pressure, the matrix modulus must exceed the value for the previous matrix model at the maximum pressure. By analysing the stress–strain curve at the guard cell mid‐point we estimated *E* ≈ 154.6 MPa, which is ≈12× higher than in the undeformed state. The same analysis in the highly stressed region at the end of the pore resulted in *E* = 116.8 MPa. The precise location of these points is shown in Figure S1. We therefore conclude that although circumferential CMFs are critical for stomata to open successfully, the cell wall matrix must stiffen significantly as pressure increases in order to provide robust mechanisms for limiting the aperture with increasing pressure.

To investigate how volume changes with pressure, we compared the volume change for the previous and the updated cell wall matrix models (Figure 3b). The guard cell volume–pressure profile is nonlinear for the stiffening cell wall matrix profile, and the sensitivity to pressure is greatest at the lower end of the pressure range. The increased sensitivity in part of the pressure range helps to explain conflicting stomatal experiments, where the guard cell volume has been separately shown to increase nonlinearly (Franks *et al*., 2001) and linearly (Shope and Mott, 2006) with increasing pressure. This highlights the importance of capturing stomatal dynamics over the full pressure range, particularly at low pressures, and helps to explain the different experimental results.

To assess the impact on the opening dynamics of changes to the elastic moduli of the cell wall components, we tested different CMF moduli with a fixed value for *G*_0_, and then we fixed the CMF modulus and varied *G*_0_ (Figure S7). When the modulus of the CMFs is fixed, an increase in the small‐strain shear modulus, *G*_0_, results in the final aperture and the guard cell volume decreasing until the matrix becomes so stiff that the change in pressure has a negligible effect and the stoma hardly deforms from its initial shape. As expected, changes to *G*_0_ hardly affect the guard cell width because hoop rigidity is present in the guard cells as a result of the strong CMFs. These results allow us to predict how opening dynamics may be linked to the stiffness of the cell wall matrix, as the model predicts that the stoma will open less when the cell wall matrix is stiffer, and open more when the matrix is less stiff.

By systematically degrading specific pectins in the cell wall, for example by enzymatic digest, one could identify pectins that significantly contribute to the stiffness of the matrix by comparing the opening dynamics of treated and untreated plants. The model can then be used to quantify the change in matrix stiffness. Indeed, the enzymatic digestion of the pectin arabinan resulted in a decrease in the aperture relative to untreated plants (Jones *et al*., 2004), an effect attributed to arabinan maintaining the ‘fluidity’ in the pectin network. Arabinan could also be viewed as making the wall softer, so that as the arabinan content decreases, upon digestion, the wall becomes stiffer. Interpreting this result with our model suggests that arabinan content in the cell wall is inversely related to the cell wall stiffness, suggesting that stomata with increased arabinan content would open more than stomata of wild‐type plants.

Regarding the sensitivity of the model to the fibre modulus, alterations to the modulus oppositely affect the aperture and the volume: a decrease in the fibre modulus leads to a decrease in the aperture, but an increase in the guard cell volume (Figure S7). This is expected, as each guard cell would lose its hoop rigidity as the fibre modulus decreases, and, eventually, the stoma would close when the fibre modulus is sufficiently small and the cell wall becomes isotropic. As the fibre modulus decreases the guard cell width increases. Interestingly, the model predicts that there is a range of fibre moduli for which the maximum aperture occurs at a pressure that lies between the minimum and maximum pressure, an effect that, as far as we know, has not been observed experimentally. Stomata with fibres of these moduli could be identified in experiments because the guard cell width and volume increase significantly. Increasing the fibre modulus towards the inferred value leads to a more open stoma and a constant guard cell width, until a limit is reached and the pore does not open wider as the fibre modulus is increased. In effect, the stoma gains no advantage by having fibres stronger than a certain value.

Using these results we can make predictions that shed light on experiments that alter those components of the fibre network that provide the guard cell with hoop rigidity, e.g. mutations to the cellulose synthase (CESA) genes or treatments that target cellulose directly. The model shows that a considerable decrease in the fibre modulus is necessary to decrease the aperture, but the stomata continue to function correctly until anisotropy is lost (Figure S7). Therefore, we expect CESA mutants that have less cellulose, or plants that have been treated with cellulase to digest the cellulose and hemicellulose crosslinks in the cell walls, would have functional stomata. This is in agreement with experimental observations of CESA mutants and cellulase‐treated plants: the stomata of the CESA mutant, *cesa* ^je5^, which has diminished cellulose content compared with the wild type, are consistently more open than the wild type in time‐series experiments, but otherwise function normally (Rui and Anderson, 2016); cellulase treatment does not impair stomatal function in several plant species (Jones *et al*., 2003; Rui and Anderson, 2016). Interestingly, Rui and Anderson (2016) found that *cesa* ^je5^ and cellulase‐treated plants opened their stomata more than wild‐type and untreated plants, respectively. Although this may seem in conflict with the model, where lower CMF strength leads to a lower aperture and wider guard cells, the guard cell width of the *cesa* ^je5^ stomata remained constant during opening, implying that the fibre strength and hence the hoop rigidity had not been significantly impaired (Figure S7). The phenomenon of increased stomatal opening is, therefore, unlikely to be the result of a decrease in fibre strength, but is most likely caused by an effect not currently captured in our model. Indeed, Rui and Anderson (2016) concluded that the increased stomatal opening resulted from the CMFs being more loosely coupled along the guard cell axis, and hence were able to move apart more easily along the guard cell axis.

### Quantifying the role of pectin in stomatal dynamics from Arabidopsis cell wall mutants

To test the model predictions, we performed experiments on wild‐type plants and three T‐DNA mutants in Arabidopsis, where each mutant is implicated in altered cell wall organization or pectin composition. The three cell wall mutants are *irx8*,* pmr5* and *pmr6*. The *irx8* mutant is deficient in the hemicellulose building block, xylose, and exhibited brittle behaviour under mechanical load (Balsamo *et al*., 2015). As xylose is related to CMF cross‐linking, the model predicts that the stomata will function normally, and possibly have an increased aperture relative to the wild type (Rui and Anderson, 2016). Both *pmr5* and *pmr6* correspond to mutations to the powdery mildew‐resistant genes, *PMR5* and *PMR6*, respectively. The *pmr5* and *pmr6* mutants show increased pectin content, notably with respect to arabinan, with *pmr6* having a significant increase relative to the wild type (Vogel *et al*., 2002, 2004). Based on our predictions related to arabinan and its effect on matrix stiffness, we expect the stomata of *pmr5* and *pmr6* to open more than in the wild type, and we predict that *pmr6* will open its stomata more than the other mutants and significantly more than the wild type.

**Figure 4:**
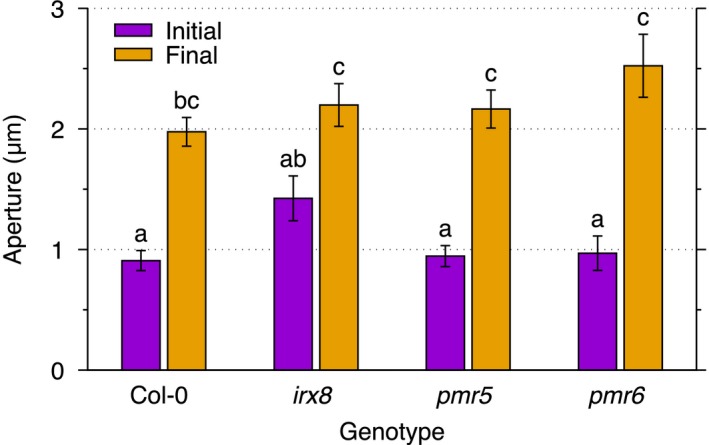
Stomatal apertures before and after the application of fusicoccin. The initial apertures were measured 5 min after the application of 50 μm fusicoccin and the final apertures were measured at 51 min after the treatment. The mean aperture and the standard error are shown for each genotype at each time point (for between eight and 13 stomata per genotype per time point in two independent experiments). Letters indicate a significant difference between mean values computed using Tukey's multiple comparison post‐hoc test with a significance level of 0.05.

We induced stomatal opening in each genotype by applying the cytotoxin fusicoccin (FC), and compared the opening dynamics with wild‐type plants. All of the genotypes respond to the FC treatment and significantly open their stomata (Figure 4). The initial aperture for the wild type and the *pmr5* and *pmr6* mutants show little difference, but *irx8* plants have stomata that are initially more open. Regarding the final aperture, the stomata of the *irx8*,* pmr5* and *pmr6* mutants are more open than the wild type, but do not significantly differ. As expected, the reduction in xylose in *irx8* does not impair stomatal function significantly, although the increased initial aperture may be the result of alterations in hemicellulose cross‐linking. The initial apertures of *pmr5* and *pmr6* are similar to that in the wild type, and the final aperture, as expected, is greater than in the wild type, with *pmr6* showing the largest increase. From the model, the increased opening of *pmr5* and *pmr6* is indicative of a decrease in the stiffness of the cell wall matrix.

Next, we used the model to quantify the change in the matrix stiffness. In the following simulations we assume that the guard cell walls of the different stomata have the same cell wall thickness and are all subject to the same pressure change. Furthermore, by only varying material parameters in the model, we are essentially asking whether different mutant behaviour – regardless of their nature – can be accounted for by a change in cell wall material. Details on the assumptions of the cell wall model are presented in the Experimental procedures. We measured the stomata in the experiments and then we initialized the model for each genotype using the mean values for the stoma length and width, together with the pore length and aperture (Table 4). Then we inflated each guard cell using an estimated range for the turgorin Arabidopsis guard cells (see Experimental procedures) and inferred the elastic moduli for the guard cell walls of each genotype; the matrix stiffnesses and fibre strengths are shown in Table 3. The inferred elastic moduli from the model show that the small‐strain shear modulus of the *pmr6* mutant is 40% lower than that of the wild type, which is a significant decrease in stiffness relative to the wild type. The robustness of these results is given in Table S1 as a function of the cell wall material parameters. Using the model to interpret experimental results thus allows for the quantification of biomechanical changes of the cell wall.

**Table 4:** Measurements from experiments and the model for Arabidopsis stomata

	Units	Closed stoma		Open stoma		Diff.
		Observed	Model	Observed	Model	
Col‐0						
Stoma length	μm	29.9 (±0.8)	29.9	29.8 (±0.7)	30.5	+0.7
Pore length	μm	–	9.4	9.4 (±0.4)	9.4	0.0
Aperture	μm	0.9 (±0.1)	0.9	2.0 (±0.1)	2.0	0.0
Guard cell width	μm	9.9 (±0.3)	9.9	10.4 (±0.3)	10.3	−0.1
*irx8*						
Stoma length	μm	30.4 (±1.1)	30.4	29.9 (±1.0)	30.7	+0.8
Pore length	μm	–	11.5	11.5 (±0.8)	11.5	0.0
Aperture	μm	1.4 (±0.2)	1.4	2.2 (±0.2)	2.2	0.0
Guard cell width	μm	10.0 (±0.3)	10.0	10.5 (±0.3)	10.3	−0.2
*pmr5*						
Stoma length	μm	29.6 (±1.1)	29.6	30.0 (±1.1)	30.3	+0.3
Pore length	μm	–	10.6	10.6 (±0.7)	10.7	+0.1
Aperture	μm	0.9 (±0.1)	0.9	2.2 (±0.2)	2.2	0.0
Guard cell width	μm	10.0 (±0.4)	10.0	10.3 (±0.5)	10.4	+0.1
*pmr6*						
Stoma length	μm	28.4 (±0.8)	28.4	29.4 (±0.7)	29.2	−0.2
Pore length	μm	–	11.1	11.1 (±0.6)	11.4	+0.3
Aperture	μm	1.0 (±0.1)	1.0	2.5 (±0.3)	2.5	0.0
Guard cell width	μm	11.0 (±0.2)	11.0	11.2 (±0.2)	11.5	+0.3

The observed measurements are the mean (±SEM) values for each genotype. The values for the closed stoma were used to construct the undeformed stomatal geometry in the model. Obtaining reliable estimates for pore length for closed stomata was problematic, and we therefore used the value for the open stomata, assuming no change (Rui and Anderson, 2016). For the closed stomata, the dorsal and ventral wall thicknesses were set to 0.5 μm, which is in the range for the epidermal cell wall thickness of young leaves (Forouzesh *et al*., 2013). The tip wall thickness was set to 0.1 μm and was estimated from Akita *et al*. (2016).

## Discussion and Conclusions

We have sought to use minimal computational models of guard cells to understand the biomechanical principles of their reversible opening and closing behaviour. Our focus has been on the dynamics of isolated guard cells. Stomata are surrounded by epidermal cells that sit on top of the mesophyll; however, many experimental results are from epidermal peels where the stomata are surrounded by ruptured epidermal cells (Spence *et al*., 1986; Franks *et al*., 2001). In Arabidopsis, guard cells often tend to sit slightly above the epidermal cells (on small ‘hills’), thereby reducing their physical interaction with neighbouring cells (McLachlan *et al*., 2014), so our isolated guard cell model may also be a useful approximation for stomata in their epidermal setting for some species. We have made a number of simplifying assumptions, and future refinements to the model could include more realistic geometries, explicitly capturing cross‐linking between the CMFs, more complex patterns of cell wall thicknesses based on experimental data and heterogeneous cell wall stiffnesses. Nevertheless, with the guard cell model presented we are able to capture existing experimental data and make a number of inferences and predictions.

By investigating the opening dynamics of a 3D biomechanical model of a guard cell, we inferred that strong, circumferential CMFs are required in the guard cell walls in order for the stoma to function correctly. Furthermore, we found that a strain‐stiffening cell wall matrix is required for the aperture to saturate with increasing pressure, and to recapture the nonlinear pressure–aperture profiles observed in pressure probe experiments. By optimising the material property parameters such that the resulting shape fits experimentally observed closed and open stomatal geometries, we were able to infer the cell wall elastic moduli. Unsurprisingly, the model predicted that the stiffness of the pectin matrix would have a noticeable effect on the opening dynamics, a feature that has been observed experimentally (Jones *et al*., 2003). Based on the model, we predict that the pectin arabinan, which has previously been implicated in altered stomatal dynamics, has an inverse relationship with the stiffness of the matrix, and that increased arabinan content leads to stomata becoming more open relative to the wild type.

Investigating the role of the cell wall in stomatal movements is challenging, however, as genetic interference with cell wall components is likely to have broader effects on plant physiology. For example, *cesa7* ^*irx3‐5*^ mutants exhibit reduced stomatal apertures, smaller guard cells and aberrant xylem function (Liang *et al*., 2010). Given that a perturbation to the cell wall is sensed as cell wall damage, and induces receptor kinase signaling (Engelsdorf and Hamann, 2014), the two processes of cell wall biomechanics and cell wall damage could interfere with each other. Indeed, cell wall damage caused by treatment with the cellulose biosynthesis inhibitor isoxaben induces the production of reactive oxygen species (Denness *et al*., 2011), which in turn could trigger the closure of stomata (McLachlan *et al*., 2014; Wolf *et al*., 2014). Thus, even with the identification of genes required for cell wall synthesis and biochemical inhibitors, it remains challenging to dissect the biomechanical function of the cell wall in guard cell dynamics. Integration of biomechanical models with signal intergration pathways may help interpret such complex behaviour.

We conclude that simple models such as the one presented here can help unravel key principles of biomechanical processes. Coupled with experimental data and optimization techniques, such models can also be used to infer cell wall material properties, interpret observed stomata shapes and predict mutant behaviour; however, given the model assumptions detailed in the text, the inference from experimental data must be used carefully, as any genetic perturbation is interpreted in the light of biomechanical properties in the model. For instance, an ion channel mutation that results in a decrease in turgor pressure, compared with the wild type, may be interpreted as a stiffer cell wall matrix. Therefore, additional information, such as pressure measurements and cell wall thickness, is required to identify the underlying cause of observed changes in guard cell dynamics.

## Experimental Procedures

### Stomatal geometry construction

Stomata are surrounded by epidermal cells and are attached to the mesophyll; however, many experimental results are from epidermal peels where the stomata are surrounded by ruptured epidermal cells (Spence *et al*., 1986; Franks *et al*., 2001). For better comparability with the available data, we therefore focused the modelling on the dynamics of isolated guard cells. We modelled the shape of the stoma as a hollow, deformed torus, with the stomatal and pore outlines approximated by ellipses (Figure S1). Each transverse cross section of the guard cell is taken to be circular, and we used a uniform thickness for the dorsal and ventral wall thicknesses in the initial, closed, state. The cell wall thickness for *V. faba* guard cells is set to 1 μm so that it falls within the range observed by Li *et al*. (2010). The thickness for *Arabidopsis thaliana* guard cells is unknown, so we used the epidermal wall thickness of 0.5 μm for young leaves (Forouzesh *et al*., 2013). The guard cells are separated from each other by solid walls at the tips, again with a prescribed thickness. Tip wall thickness was observed by Meckel *et al*. (2007) to be 0.3 μm for *V. faba*, and we estimated it to be 0.1 μm for Arabidopsis from figure 2(d) in Akita *et al*. (2016). The model was therefore constructed from the following geometrical parameters: (i) stomatal length and width; (ii) pore length and width; and (iii) ventral, dorsal and tip wall thicknesses, which are all in the equatorial plane of the stoma (i.e. the *x*–*y* plane) and specified in μm. The dorsal and ventral wall thicknesses define offsets to the dorsal and ventral ellipses, thus producing two further curves that represent the inner walls of the guard cells. The line joining the major axes of the dorsal and ventral ellipses defines the tip wall with its own thickness. We then divide the dorsal and ventral ellipses into an equal number of sections of equal arc length using the incomplete elliptic integral of the second kind. We then generated each cross section by rotating each pair of dorsal–ventral wall points about their half‐way point, and in the vertical plane containing the points. We imposed symmetry conditions on the stoma by allowing the points that initially lie in each of the *x*–*y*,* x*–*z* and *y*–*z* planes to move, but constraining them to remain in the symmetry plane. A stencil for the model stoma together with views of the outside and inside of a model guard cell are shown in Figure S1, where the elliptical outlines of the stoma and the pore together with the uniform cell wall thicknesses can be seen. We constructed the closed stoma using the lengths and widths of the pore and the stoma, together with the cell wall thicknesses. The dimensions for the stomata are given in Tables 1 and 4.

### Turgor pressure

Turgor pressure, *P*, is the difference between the pressures on the inside and on the outside of cells. We chose *P* as the independent variable in our simulations, which we applied to the inner surface of each guard cell. We increased *P* from 0 to 5 MPa, so that the minimum and maximum pressures are associated with closed and fully open stomata, respectively, in line with pressure probe experiments in *V. faba* (Franks *et al*., 1998, 2001). We used the same pressure range for the stomata of *A. thaliana*.

### The cell wall model

We used a two‐phase hyperelastic strain energy density function, *W*, to model the stress–strain relationship in the guard cell wall (Chaplain, 1993). This approach has been used for modelling plant growth (Geitmann and Ortega, 2009). Relations of this kind can be used to approximate elastic, visco‐elastic and plastic deformations, with Hooke's law being an example applicable to linear elasticity. As stomata reversibly open and close, we approximated the cell wall behaviour as being elastic. We chose an elastic model for the cell wall that is well suited to the three‐dimensional nature of the morphology of the stoma, and which can also incorporate the anisotropy contributed to the cell wall by the circumferentially oriented CMFs. To separate the isotropic cell wall matrix from the CMFs we used an uncoupled formulation so that:

$$W=W_{\mathrm{matrix}}+W_{\mathrm{CMFs}}$$

where we chose *W*_matrix_ to be either the Mooney–Rivlin (Rivlin, 1948) or the Veronda–Westmann model (Veronda and Westmann, 1970), where the latter explicitly incorporates stiffening. Both models for the matrix are mathematically characterized by two parameters, which can be brought together to form the small‐strain shear modulus, *G*_0_, permitting physical comparison of the values. The fibre contribution to the energy, *W*_CMFs_,, is independent of the choice for *W*_matrix_. By uncoupling the matrix from the fibres in this way we are able to separately examine the contribution of each component to the dynamics of stomatal opening. We chose the open‐source and freely available Finite Elements for Biomechanics software (febio; Maas *et al*., 2012) for our simulations, which is a set of tools specifically developed for biomechanical investigations. In this software, the models are characterized by eight parameters: three for the isotropic cell wall matrix and five for the CMFs. The three cell wall matrix parameters are C_1_, C_2_ and the bulk modulus, *K*, but we reduce this number to two by assuming that the cell wall is incompressible, which is achieved by setting *K* = 10 GPa, resulting in a Poisson's ratio of ≈0.5. The small‐strain shear modulus, *G*_0_, of the cell wall matrix is related to *C*_1_ and *C*_2_ by *G*_0_ = *C*_1_ × *C*_2_ and *G*_0_ = 2(*C*_1_ + *C*_2_) for the Veronda–Westmann and the Mooney–Rivlin models, respectively. Additionally, for an incompressible material the Young's modulus, *E*, is related to *G* by *E* = 3*G*. These expressions can be used to get estimates for the small‐strain shear modulus and the Young's modulus of the cell wall matrix, although *G* and hence *E* will only be strictly valid for small stretches. We also use the gradient of the effective stress‐strain curve at selected points, the locations of which are shown in Figure S1, to estimate maximal values of *E*. The parameters of the cell wall model, therefore, dictate how changes in the pressure translate into changes in the geometry.

The fibre model is divided into three regions according to whether the embedding material is compressed, stretched (but the fibres are not yet straight) and when the fibres are straight, i.e. the fibres are permitted to be non‐straight when the embedding matrix is unstretched; full details are given in the febio documentation. The five fibre parameters are *C*_3_, *C*_4_, *C*_5_, λ_m_ and a unit vector that defines the fibre direction. The first two fibre parameters pertain to stretched but unstraightened fibres, and define a scaling factor for the stresses and a straightening rate, respectively. The *C*_5_ parameter is the modulus of straightened fibres and λ_m_ is the stretch (in the fibre direction) of the cell wall matrix at which the fibres become straight. In guard cells the embedded CMFs are well known to lie in a radial pattern, so that they travel circumferentially around the guard cell cross section (e.g. Fujita and Wasteneys, 2013), and so we set the fibre direction accordingly. We also included circumferential CMFs in the tip wall: the fibre direction is shown in Figure S1. In our simulations, we set λ_m_ = 1 because values greater than this led to the guard cell width increasing, which is not observed experimentally. This choice for λ_m_ removes *C*_3_ and *C*_4_ from the parameterisation, leaving *C*_5_ to be determined. We did not ascribe a spatial dependence to the cell wall parameters, although this could be incorporated if required. Finally, as the starting spatial distribution of mechanical stress is unknown, we initialized the model stoma in the closed state, which we associate with zero turgor pressure, and set the stress and strain in the cell wall to zero everywhere.

### Finite element mesh construction and validation

To prepare the geometry for the finite‐element method we require a suitable mesh. We chose a mesh defined in terms of hexahedral and pentahedral prismatic elements. Dividing each guard cell into a total of around 20 000 elements was sufficient to ensure that the mesh resolution did not affect the results. The mesh quality was checked using the condition number metric in vtk (http://www.vtk.org) The model construction was performed by a custom python script (http://www.python.org), which also exported the geometry, fibre directions, cell wall material parameters, boundary conditions and pressure curve to a file in the format required by febio (Maas *et al*., 2012). The results of the simulation were written to a binary file by febio, which was processed by another custom python script that tracked various quantities over the pressure range. At each step of the simulation, the stomatal length and width, pore length and width, and the pore area, together with the surface area and enclosed volume of each guard cell, were recorded; the algorithm for the volume calculation is described in (Mirtich, 1996).

### Cell wall parameter inference

In general, material parameters are found by fitting an elastic model to an experimentally determined stress–strain curve for a sample of the material (e.g. Martins *et al*., 2006). Here we used the stomatal geometry to infer the cell wall moduli. To find the set of material parameters that produced the best match to the open stoma we quantified the difference between the model and observation using the distance (Tofallis, 2014):

$$d=\sum_{i}{\left(\text{ln}\left(M_{i}/E_{i}\right)\right)}^{2}$$

where for each of the *i* measurements, *M*_i_ and *E*_i_ correspond to the value produced by the model and the experimentally observed value, respectively. We used stomatal length, guard cell width, pore length and aperture in the metric. Optimization was performed using the ‘SLSQP’ method in the python package scipy to determine cell wall parameters that provide the best fit to the experimental data according to the distance *d*. The code will be made publically available to coincide with publication.

### Plant materials and growth conditions

The *A. thaliana* wild type (Col‐0) as well as T‐DNA mutants *irx8* (At5g54690), *pmr5* (At5g58600) and *pmr6* (At3g54920) were used in this work. Plants were grown on soil for 4 weeks under 10 h light/14 h dark at 22°C/20°C, respectively, and 65% humidity.

### Stomatal opening treatment and imaging

Four‐week‐old leaves at the same developmental stage were harvested in the morning after a minimum of 3 h of light and transferred in a buffer [50 mm KCl, 10 μm CaCl_2_, 10 mm 2‐(*N*‐morpholino)ethanesulfonic acid (MES), pH 6.1, 0.01% Tween] for 3 h in the dark at room temperature to induce stomatal closure. The samples were then treated with 50 μm fusicoccin (Sigma‐Aldrich, https://www.sigmaaldrich.com) in the same buffer. All images were acquired with a bright‐field microscope DM‐R (Leica, http://www.leica.com) at 40× magnification. Stomatal aperture was measured at each time point from light microscopy pictures using imagej (Schneider *et al*., 2012). The overall stomatal length and width were measured at the start point and end point using imagej. An average of 11.5 stomata per genotype were measured for 1 h in two independent experiments. Stomata that did not respond to fusicoccin were excluded from the analysis.

## Code availability

Finite‐element modelling was performed within the febio framework (http://www.febio.org) . All of the scripts used to run these simulations, process the data and generate the graphs are freely available at https://github.com/woolfeh/stomasimulator.

## Funding

We acknowledge funding from the Biotechnology and Biological Sciences Research Council (BBSRC) and from an ERC Starting Grant (SR). Research at Molina lab was supported by grant BIO2012‐32910 from Spanish Ministery of Economy and Competitiveness (MINECO). The funders had no role in study design, data collection and interpretation, or the decision to submit the work for publication.

## Author contributions

H.C.W. performed the computational modelling and python scripting; M.K. designed the experiments; G.B. performed the experiments; G.B. and H.C.W performed the image analysis; R.J.M., S.R. and H.C.W. designed the project. E.M. characterized the cell wall mutants. H.C.W., R.J.M. and S.R. wrote the manuscript, with contributions from G.B., M.K. and A.M.

## Conflict of interest

The authors have no conflict of interest to declare.

## Supporting information


**Figure S1.** Geometry of the model stoma.
**Figure S2.** Cell wall thickness and matrix stiffness do not qualitatively affect stomatal function.
**Figure S3.** Guard cell aspect ratio only affects stomatal dynamics at low pressures.
**Figure S4.** Stress and strain directions show hoop rigidity and longitudinal guard cell lengthening.
**Figure S5.** Circumferentially oriented cellulose fibres are critical for proper stomatal function.
**Figure S6.** Stomatal opening induces stress and strain hot spots in guard cell walls.
**Figure S7.** Aperture is oppositely affected by a stiffer cell wall matrix versus stiffer CMFs.

**Table S1.** Change in stomata geometry of the cell wall genotypes of Arabidopsis as a function of the cell wall material property parameters.

## References

[tpj13640-bib-0001] Akita, K. , Kobayashi, M. , Sato, M. , Kutsuna, N. , Ueda, T. , Toyooka, K. , Nagata, N. , Hasezawa, S. and Higaki, T. (2016) Cell wall accumulation of fluorescent proteins derived from a trans‐Golgi cisternal membrane marker and paramural bodies in interdigitated Arabidopsis leaf epidermal cells. Protoplasma, 254, 367–377. 26960821 10.1007/s00709-016-0955-1

[tpj13640-bib-0002] Amsbury, S. , Hunt, L. , Elhaddad, N. ***et al*** (2016) Stomatal function requires pectin de‐methyl‐esterification of the guard cell wall. Curr. Biol. 26, 2899–2906. 27720618 10.1016/j.cub.2016.08.021 PMC5106435

[tpj13640-bib-0003] Aylor, D.E. , Parlange, J.‐Y. and Krikorian, A.D. (1973) Stomatal mechanics. Am. J. Bot. 60, 163–171.

[tpj13640-bib-0004] Balsamo, R. , Boak, M. , Nagle, K. , Peethambaran, B. and Layton, B. (2015) Leaf biomechanical properties in Arabidopsis thaliana polysaccharide mutants affect drought survival. J. Biomech. 48, 4124–4129. 26520913 10.1016/j.jbiomech.2015.10.016

[tpj13640-bib-0005] Bargel, H. , Spatz, H.‐C. , Speck, T. and Neinhuis, C. (2004) Two‐dimensional tension tests in plant biomechanics–sweet cherry fruit skin as a model system. Plant Biol. 6, 432–439. 15248126 10.1055/s-2004-821002

[tpj13640-bib-0006] Beauzamy, L. , Derr, J. and Boudaoud, A. (2015) Quantifying hydrostatic pressure in plant cells by using indentation with an atomic force microscope. Biophys. J. 108, 2448–2456. 25992723 10.1016/j.bpj.2015.03.035 PMC4457008

[tpj13640-bib-0007] Bozorg, B. , Krupinski, P. and Jönsson, H. (2014) Stress and strain provide positional and directional cues in development. PLoS Comput. Biol. 10, e1003410. 24415926 10.1371/journal.pcbi.1003410 PMC3886884

[tpj13640-bib-0008] Braybrook, S.A. , Hofte, H. and Peaucelle, A. (2012) Probing the mechanical contributions of the pectin matrix: insights for cell growth. Plant. Signal. Behav. 7, 1037–1041. 22836501 10.4161/psb.20768 PMC3474675

[tpj13640-bib-0009] Chanliaud, E. , Burrows, K. , Jeronimidis, G. and Gidley, M. (2002) Mechanical properties of primary plant cell wall analogues. Planta, 215, 989–996. 12355159 10.1007/s00425-002-0783-8

[tpj13640-bib-0010] Chaplain, M. (1993) The strain energy function of an ideal plant cell wall. J. Theor. Biol. 163, 77–97.

[tpj13640-bib-0011] Cheng, Q. and Wang, S. (2008) A method for testing the elastic modulus of single cellulose fibrils via atomic force microscopy. Compos. Part A: Appl. Sci. Manufac. 39, 1838–1843.

[tpj13640-bib-0012] Cooke, J.R. , DeBaerdemaeker, J.G. , Rand, R.H. and Mang, H.A. (1976) A finite element shell analysis of guard cell deformations. Trans. ASAE 19, 1107.

[tpj13640-bib-0013] Cosgrove, D.J. (1988) In defence of the cell volumetric elastic modulus. Plant Cell Environ. 11, 67–69. 11542201

[tpj13640-bib-0014] Cosgrove, D.J. (1993) Wall extensibility: its nature, measurement and relationship to plant cell growth. New Phytol. 124, 1–23. 11537718 10.1111/j.1469-8137.1993.tb03795.x

[tpj13640-bib-0015] Cosgrove, D.J. (2016) Plant cell wall extensibility: connecting plant cell growth with cell wall structure, mechanics, and the action of wall‐modifying enzymes. J. Exp. Bot. 67, 463–476. 26608646 10.1093/jxb/erv511

[tpj13640-bib-0016] DeMichele, D.W. and Sharpe, P.J.H. (1973) Analysis of mechanics of guard cell motion. J. Theor. Biol. 41, 77–96. 4754908 10.1016/0022-5193(73)90190-2

[tpj13640-bib-0017] Denness, L. , McKenna, J.F. , Segonzac, C. , Wormit, A. , Madhou, P. , Bennett, M. , Mansfield, J. , Zipfel, C. and Hamann, T. (2011) Cell wall damage‐induced lignin biosynthesis is regulated by a reactive oxygen species‐ and jasmonic acid‐dependent process in Arabidopsis. Plant Physiol. 156, 1364–1374. 21546454 10.1104/pp.111.175737 PMC3135913

[tpj13640-bib-0018] Dumais, J. , Shaw, S.L. , Steele, C.R. , Long, S.R. and Ray, P.M. (2006) An anisotropic‐viscoplastic model of plant cell morphogenesis by tip growth. Int. J. Dev. Biol. 50, 209–222. 16479489 10.1387/ijdb.052066jd

[tpj13640-bib-0019] Engelsdorf, T. and Hamann, T. (2014) An update on receptor‐like kinase involvement in the maintenance of plant cell wall integrity. Ann. Bot. 114, 1339–1347. 24723447 10.1093/aob/mcu043 PMC4195549

[tpj13640-bib-0020] Forouzesh, E. , Goel, A. , Mackenzie, S.A. and Turner, J.A. (2013) In vivo extraction of Arabidopsis cell turgor pressure using nanoindentation in conjunction with finite element modeling. Plant J. 73, 509–520. 23036157 10.1111/tpj.12042

[tpj13640-bib-0021] Franks, P.J. (2003) Use of the pressure probe in studies of stomatal function. J. Exp. Bot. 54, 1495–1504. 12730269 10.1093/jxb/erg162

[tpj13640-bib-0022] Franks, P.J. and Farquhar, G.D. (2007a) The mechanical diversity of stomata and its significance in gas‐exchange control. Plant Physiol. 143, 78–87. 17114276 10.1104/pp.106.089367 PMC1761988

[tpj13640-bib-0023] Franks, P.J. , Cowan, I.R. and Farquhar, G.D. (1998) A study of stomatal mechanics using the cell pressure probe. Plant Cell Environ. 21, 94–100.

[tpj13640-bib-0024] Franks, P.J. , Buckley, T.N. , Shope, J.C. and Mott, K.A. (2001) Guard cell volume and pressure measured concurrently by confocal microscopy and the cell pressure probe. Plant Physiol. 125, 1577–1584. 11299339 10.1104/pp.125.4.1577 PMC88815

[tpj13640-bib-0025] Fujita, M. and Wasteneys, G.O. (2013) A survey of cellulose microfibril patterns in dividing, expanding, and differentiating cells of *Arabidopsis thaliana* . Protoplasma 251, 687–698. 24169947 10.1007/s00709-013-0571-2

[tpj13640-bib-0026] Gall, L. , Stan, R.C. , Kress, A. , Hertel, B. , Thiel, G. and Meckel, T. (2010) Fluorescent detection of fluid phase endocytosis allows for *in vivo* estimation of endocytic vesicle sizes in plant cells with sub‐diffraction accuracy. Traffic 11, 548–559. 20136778 10.1111/j.1600-0854.2010.01037.x

[tpj13640-bib-0027] Gao, X.‐Q. , Li, C.‐G. , Wei, P.‐C. , Zhang, X.‐Y. , Chen, J. and Wang, X.‐C. (2005) The dynamic changes of tonoplasts in guard cells are important for stomatal movement in Vicia faba. Plant Physiol. 139, 1207–1216. 16244153 10.1104/pp.105.067520 PMC1283759

[tpj13640-bib-0028] Geitmann, A. and Ortega, J.K.E. (2009) Mechanics and modeling of plant cell growth. Trends Plant Sci. 14, 467–478. 19717328 10.1016/j.tplants.2009.07.006

[tpj13640-bib-0029] Gibson, L.J. (2012) The hierarchical structure and mechanics of plant materials. J. R. Soc. Interface 9, 2749–2766. 22874093 10.1098/rsif.2012.0341 PMC3479918

[tpj13640-bib-0030] Hayot, C.M. , Forouzesh, E. , Goel, A. , Avramova, Z. and Turner, J.A. (2012) Viscoelastic properties of cell walls of single living plant cells determined by dynamic nanoindentation. J. Exp. Bot. 63, 2525–2540. 22291130 10.1093/jxb/err428 PMC3346220

[tpj13640-bib-0031] Heisler, M.G. , Hamant, O. , Krupinski, P. , Uyttewaal, M. , Ohno, C. , Jönsson, H. , Traas, J. and Meyerowitz, E.M. (2010) Alignment between PIN1 polarity and microtubule orientation in the shoot apical meristem reveals a tight coupling between morphogenesis and auxin transport. PLoS Biol. 8, e1000516. 20976043 10.1371/journal.pbio.1000516 PMC2957402

[tpj13640-bib-0032] Hofhuis, H. , Moulton, D. , Lessinnes, T. ***et al*** (2016) Morphomechanical innovation drives explosive seed dispersal. Cell, 166, 222–233. 27264605 10.1016/j.cell.2016.05.002 PMC4930488

[tpj13640-bib-0033] Jones, L. , Milne, J.L. , Ashford, D. and McQueen‐Mason, S.J. (2003) Cell wall arabinan is essential for guard cell function. Proc. Natl Acad. Sci. USA 100, 11783–11788. 13130074 10.1073/pnas.1832434100 PMC208835

[tpj13640-bib-0034] Jones, L. , Milne, J.L. , Ashford, D. , McCann, M.C. and McQueen‐Mason, S.J. (2004) A conserved functional role of pectic polymers in stomatal guard cells from a range of plant species. Planta, 221, 255–264. 15578215 10.1007/s00425-004-1432-1

[tpj13640-bib-0035] Kierzkowski, D. , Nakayama, N. , Routier‐Kierzkowska, A.L. , Weber, A. , Bayer, E. , Schorderet, M. , Reinhardt, D. , Kuhlemeier, C. and Smith, R.S. (2012) Elastic domains regulate growth and organogenesis in the plant shoot apical meristem. Science, 335, 1096–1099. 22383847 10.1126/science.1213100

[tpj13640-bib-0036] Kim, T.‐H. , Böhmer, M. , Hu, H. , Nishimura, N. and Schroeder, J.I. (2010) Guard cell signal transduction network: advances in understanding abscisic acid, CO2, and Ca2+ signaling. Annu. Rev. Plant Biol. 61(61), 561–591. 20192751 10.1146/annurev-arplant-042809-112226 PMC3056615

[tpj13640-bib-0037] Kollist, H. , Nuhkat, M. and Roelfsema, M.R.G. (2014) Closing gaps: linking elements that control stomatal movement. New Phytol. 203, 44–62. 24800691 10.1111/nph.12832

[tpj13640-bib-0038] Li, B. , Liu, G. , Deng, Y. ***et al*** (2010) Excretion and folding of plasmalemma function to accommodate alterations in guard cell volume during stomatal closure in *Vicia faba* L. J. Exp. Bot. 61, 3749–3758. 20603284 10.1093/jxb/erq197 PMC2921211

[tpj13640-bib-0039] Liang, Y.K. , Xie, X. , Lindsay, S.E. , Wang, Y.B. , Masle, J. , Williamson, L. , Leyser, O. and Hetherington, A.M. (2010) Cell wall composition contributes to the control of transpiration efficiency in *Arabidopsis thaliana* . Plant J. 64, 679–686. 21070419 10.1111/j.1365-313X.2010.04362.x

[tpj13640-bib-0040] Maas, S.A. , Ellis, B.J. , Ateshian, G.A. and Weiss, J.A. (2012) FEBio: finite elements for biomechanics. J. Biomech. Eng. 134, 011005. 22482660 10.1115/1.4005694 PMC3705975

[tpj13640-bib-0041] Martins, P.A.L.S. , Natal Jorge, R.M. and Ferreira, A.J.M. (2006) A comparative study of several material models for prediction of hyperelastic properties: application to silicone‐rubber and soft tissues. Strain, 42, 135–147.

[tpj13640-bib-0042] McLachlan, D.H. , Kopischke, M. and Robatzek, S. (2014) Gate control: guard cell regulation by microbial stress. New Phytol. 203, 1049–1063. 25040778 10.1111/nph.12916

[tpj13640-bib-0043] Meckel, T. , Hurst, A.C. , Thiel, G. and Homann, U. (2004) Endocytosis against high turgor: intact guard cells of *Vicia faba* constitutively endocytose fluorescently labelled plasma membrane and GFP‐tagged K+‐channel KAT1. Plant J. 39, 182–193. 15225284 10.1111/j.1365-313X.2004.02119.x

[tpj13640-bib-0044] Meckel, T. , Gall, L. , Semrau, S. , Homann, U. and Thiel, G. (2007) Guard cells elongate: relationship of volume and surface area during stomatal movement. Biophys. J. 92, 1072–1080. 17098796 10.1529/biophysj.106.092734 PMC1779957

[tpj13640-bib-0045] Mirtich, B. (1996) Fast and accurate computation of polyhedral mass properties. J. Graphics Tools 1, 31–50.

[tpj13640-bib-0046] Morris, C.E. and Homann, U. (2001) Cell surface area regulation and membrane tension. J. Membr. Biol. 179, 79–102. 11220366 10.1007/s002320010040

[tpj13640-bib-0047] Nezhad, A.S. , Naghavi, M. , Packirisamy, M. , Bhat, R. and Geitmann, A. (2013a) Quantification of cellular penetrative forces using lab‐on‐a‐chip technology and finite element modeling. Proc. Natl Acad. Sci. USA 110, 8093–8098. 23630253 10.1073/pnas.1221677110 PMC3657807

[tpj13640-bib-0048] Nezhad, A.S. , Naghavi, M. , Packirisamy, M. , Bhat, R. and Geitmann, A. (2013b) Quantification of the Young's modulus of the primary plant cell wall using Bending‐Lab‐On‐Chip (BLOC). Lab Chip 13, 2599–2608. 23571308 10.1039/c3lc00012e

[tpj13640-bib-0049] Niklas, K.J. (1992) Plant Biomechanics. Chicago: University Of Chicago Press

[tpj13640-bib-0050] Palevitz, B.A. and Hepler, P.K. (1976) Cellulose microfibril orientation and cell shaping in developing guard cells of Allium: The role of microtubules and ion accumulation. Planta 132, 71–93. 24424910 10.1007/BF00390333

[tpj13640-bib-0051] Rivlin, R.S. (1948) Large elastic deformations of isotropic materials. IV. Further developments of the general theory. Philos. Trans. R. Soc. A: Math. Phys. Eng. Sci. 241, 379–397.

[tpj13640-bib-0052] Rui, Y. and Anderson, C.T. (2016) Functional analysis of cellulose and xyloglucan in the walls of stomatal guard cells of arabidopsis. Plant Physiol. 170, 1398–1419. 26729799 10.1104/pp.15.01066 PMC4775103

[tpj13640-bib-0053] Ryden, P. , Sugimoto‐Shirasu, K. , Smith, A.C. , Findlay, K. , Reiter, W.‐D. and McCann, M.C. (2003) Tensile properties of Arabidopsis cell walls depend on both a xyloglucan cross‐linked microfibrillar network and rhamnogalacturonan II‐borate complexes. Plant Physiol. 132, 1033–1040. 12805631 10.1104/pp.103.021873 PMC167041

[tpj13640-bib-0054] Sampathkumar, A. , Krupinski, P. , Wightman, R. , Milani, P. , Berquand, A. , Boudaoud, A. , Hamant, O. , Jönsson, H. and Meyerowitz, E.M. (2014) Subcellular and supracellular mechanical stress prescribes cytoskeleton behavior in Arabidopsis cotyledon pavement cells. eLife, 3, e01967. 24740969 10.7554/eLife.01967 PMC3985187

[tpj13640-bib-0055] Schneider, C.A. , Rasband, W.S. and Eliceiri, K.W. (2012) NIH Image to ImageJ: 25 years of image analysis. Nat. Methods 9, 671–675. 22930834 10.1038/nmeth.2089 PMC5554542

[tpj13640-bib-0056] Shope, J.C. and Mott, K.A. (2006) Membrane trafficking and osmotically induced volume changes in guard cells. J. Exp. Bot. 57, 4123–4131. 17088361 10.1093/jxb/erl187

[tpj13640-bib-0057] Somerville, C. , Bauer, S. , Brininstool, G. ***et al*** (2004) Toward a systems approach to understanding plant cell walls. Science, 306, 2206–2211. 15618507 10.1126/science.1102765

[tpj13640-bib-0058] Spence, R.D. , Wu, H.‐I. , Sharpe, P.J.H. and Clark, K.G. (1986) Water stress effects on guard cell anatomy and the mechanical advantage of the epidermal cells. Plant Cell Environ. 9, 197–202.

[tpj13640-bib-0059] Tofallis, C. (2014) A better measure of relative prediction accuracy for model selection and model estimation. J. Oper. Res. Soc. 66, 1352–1362.

[tpj13640-bib-0060] Toole, G.A. , Gunning, P.A. , Parker, M.L. , Smith, A.C. and Waldron, K.W. (2001) Fracture mechanics of the cell wall of Chara corallina. Planta, 212, 606–611. 11525518 10.1007/s004250000425

[tpj13640-bib-0061] Veronda, D.R. and Westmann, R.A. (1970) Mechanical characterization of skin—Finite deformations. J. Biomech. 3, 111–124. 5521524 10.1016/0021-9290(70)90055-2

[tpj13640-bib-0062] Vogel, J.P. , Raab, T.K. , Schiff, C. and Somerville, S.C. (2002) PMR6, a pectate lyase‐like gene required for powdery mildew susceptibility in Arabidopsis. Plant Cell. 14, 2095–2106. 12215508 10.1105/tpc.003509 PMC150758

[tpj13640-bib-0063] Vogel, J.P. , Raab, T.K. , Somerville, C.R. and Somerville, S.C. (2004) Mutations in PMR5 result in powdery mildew resistance and altered cell wall composition. Plant J. 40, 968–978. 15584961 10.1111/j.1365-313X.2004.02264.x

[tpj13640-bib-0064] Vogler, H. , Felekis, D. , Nelson, B. and Grossniklaus, U. (2015) Measuring the mechanical properties of plant cell walls. Plants, 4, 167–182. 27135321 10.3390/plants4020167 PMC4844320

[tpj13640-bib-0065] Webb, A.A.R. , Larman, M.G. , Montgomery, L.T. , Taylor, J.E. and Hetherington, A.M. (2001) The role of calcium in ABA‐induced gene expression and stomatal movements. Plant J. 26, 351–362. 11439123 10.1046/j.1365-313x.2001.01032.x

[tpj13640-bib-0066] Weber, A. , Braybrook, S. , Huflejt, M. , Mosca, G. , Routier‐Kierzkowska, A.‐L. and Smith, R.S. (2015) Measuring the mechanical properties of plant cells by combining micro‐indentation with osmotic treatments. J. Exp. Bot. 66, 3229–3241. 25873663 10.1093/jxb/erv135 PMC4449541

[tpj13640-bib-0067] Wolf, S. , van der Does, D. , Ladwig, F. ***et al*** (2014) A receptor‐like protein mediates the response to pectin modification by activating brassinosteroid signaling. Proc. Natl Acad. Sci. 111, 15261–15266. 25288746 10.1073/pnas.1322979111 PMC4210321

[tpj13640-bib-0068] Wolfe, J. , Dowgert, M.F. and Steponkus, P.L. (1986) Mechanical study of the deformation and rupture of the plasma membranes of protoplasts during osmotic expansions. J. Membr. Biol. 93, 63–74.

[tpj13640-bib-0069] Wu, H.‐I. and Sharpe, P.J.H. (1979) Stomatal mechanics II*: material properties of guard cell walls. Plant Cell Environ. 2, 235–244.

[tpj13640-bib-0070] Zamil, M.S. , Yi, H. , Haque, M.A. and Puri, V.M. (2013) Characterizing microscale biological samples under tensile loading: stress‐strain behavior of cell wall fragment of onion outer epidermis. Am. J. Bot. 100, 1105–1115. 23720433 10.3732/ajb.1200649

