# Supplementary material for: A computational approach for inferring the cell wall properties that govern guard cell dynamics
Source: Plant J. 2017 Aug 23;92(1):5–18. doi: 10.1111/tpj.13640 (PMC5637902; doi:10.1111/tpj.13640)
Supplement: Supplementary file 1 — Figure S1. Geometry of the model stoma. Figure S2. Cell wall thickness and matrix stiffness do not qualitatively affect stomatal function. Figure S3. Guard cell aspect ratio only affects stomatal dynamics at low pressures. Figure S4. Stress and strain directions show hoop rigidity and longitudinal guard cell lengthening. Figure S5. Circumferentially oriented cellulose fibres are critical for proper stomatal function. Figure S6. Stomatal opening induces stress and strain hot spots in guard cell walls. Figure S7. Aperture is oppositely affected by a stiffer cell wall matrix versus stiffer CMFs. [file TPJ-92-5-s001.pdf]

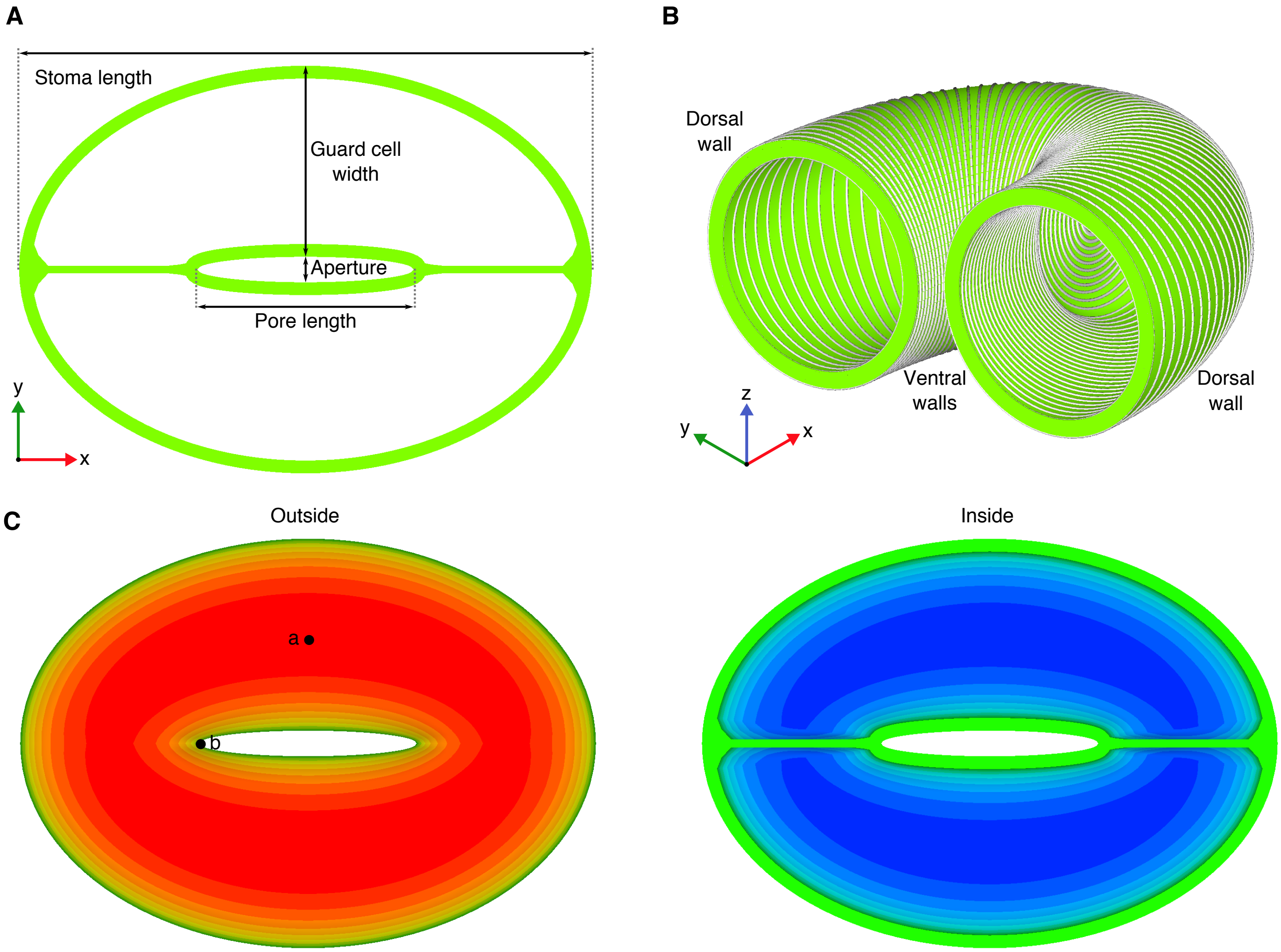

Figure S1. Geometry of the model stoma. **A**, Template for the equatorial plane of the model stoma indicating the stoma length, guard cell width, aperture (or pore width) and pore length measurements. **B**, Perspective view of half of the model stoma showing the circumferential fibres (white lines). **C**, Views of the model stoma from above (positive z) showing the outside (left panel) and the inside of the stoma after the removal of the upper periclinal wall to show the cell wall thicknesses (right panel). Young's moduli are estimated from the stress-strain curves at the points labelled **a** and **b**. Positive and negative z are coloured red and blue, respectively. The stomatal dimensions are given in Table 1.

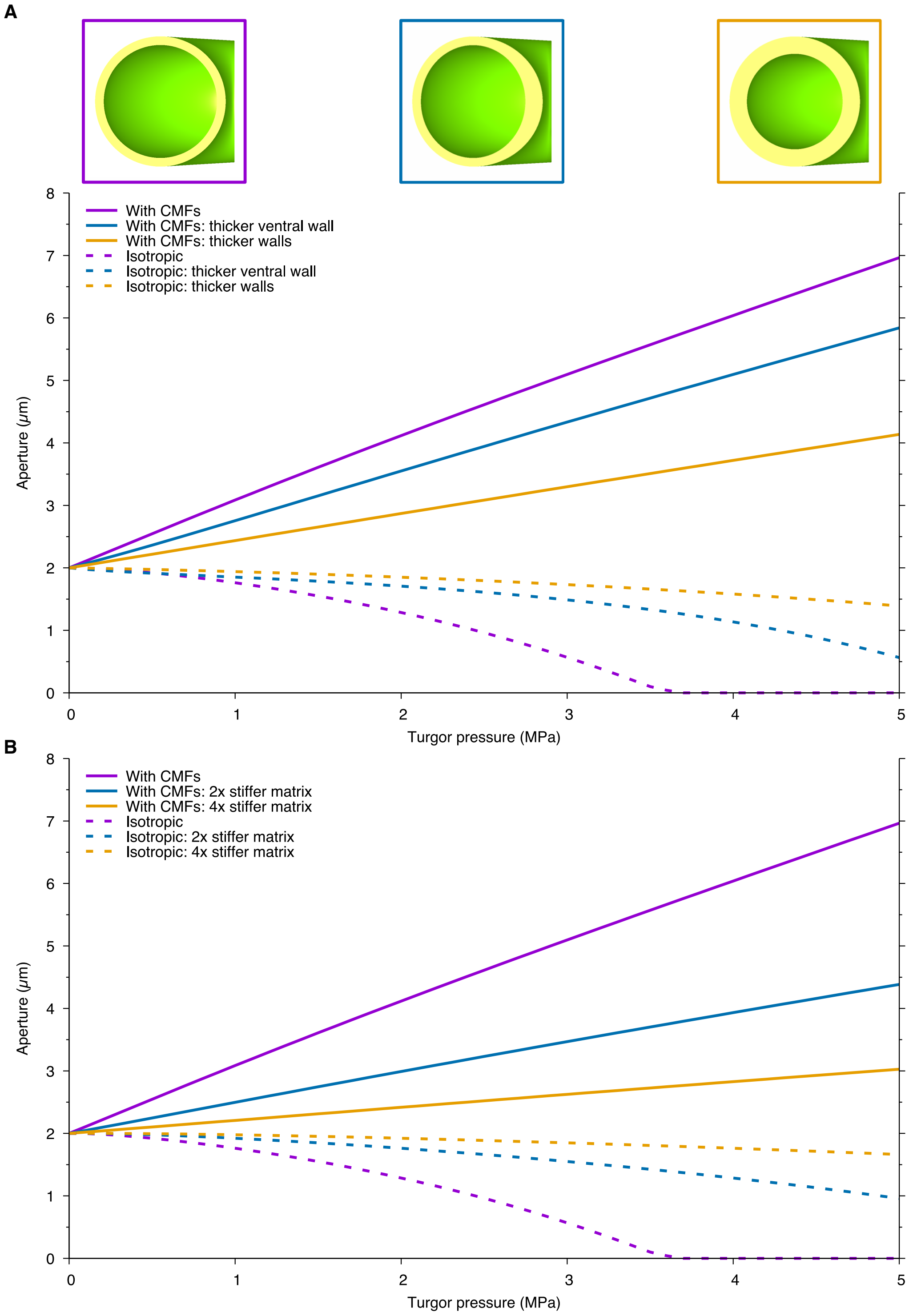

Figure S2. Cell wall thickness and matrix stiffness do not qualitatively affect stomatal function. Turgor pressure vs. aperture for a stoma with dimensions given in Table 1. **A**, Aperture profiles for the stomata in Fig. 1 together with profiles for a stoma with a relatively thicker ventral wall, and a stoma with a uniformly thicker cell wall. Single guard cell cross-sections for the models are shown in the top panel. **B**, Aperture profiles for the same stomata but with the stiffness of the cell wall matrix doubled and quadrupled. The cell wall parameters are either the 'Isotropic' values (dashed lines) or the 'With CMFs' values (solid lines) listed in Table 2.

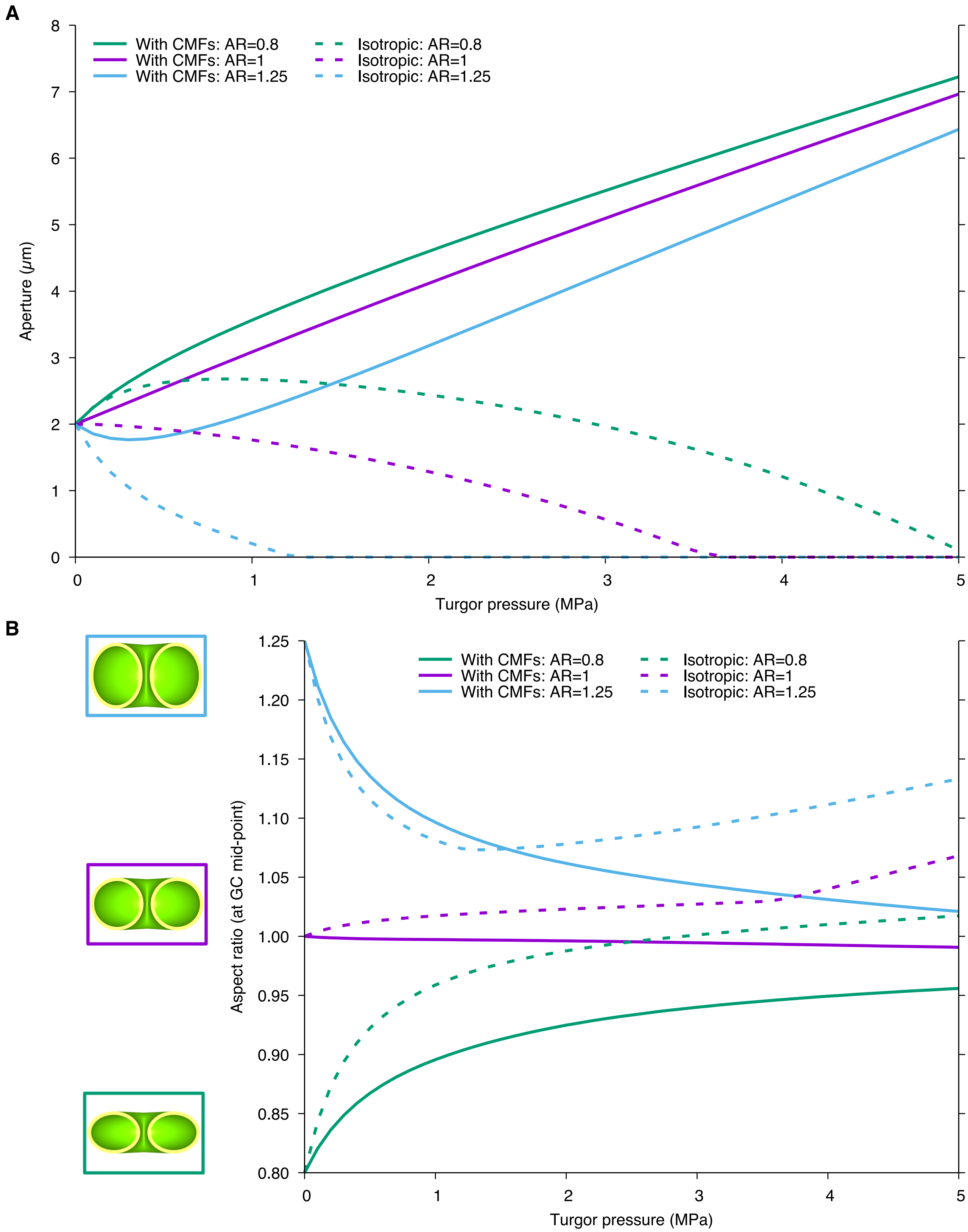

Figure S3. Guard cell aspect ratio only affects stomatal dynamics at low pressures. **A**, Stomata with guard cells that have circular or flattened cross-sections close as pressure increases when the cell wall is isotropic (parameters are ‘Isotropic’ in Table 1) and open when CMFs are present. The aspect ratio (AR) is the guard cell depth (into the leaf) divided by the guard cell width at the mid-point. **B**, The flattened guard cells rapidly lose their eccentric cross-section. The cross-sections of the stomata are shown on the left for each aspect ratio. The stomatal dimensions are given in Table 1, and the cell wall parameters are either the ‘Isotropic’ values (dashed lines) or the ‘With CMFs’ values (solid lines) listed in Table 2.

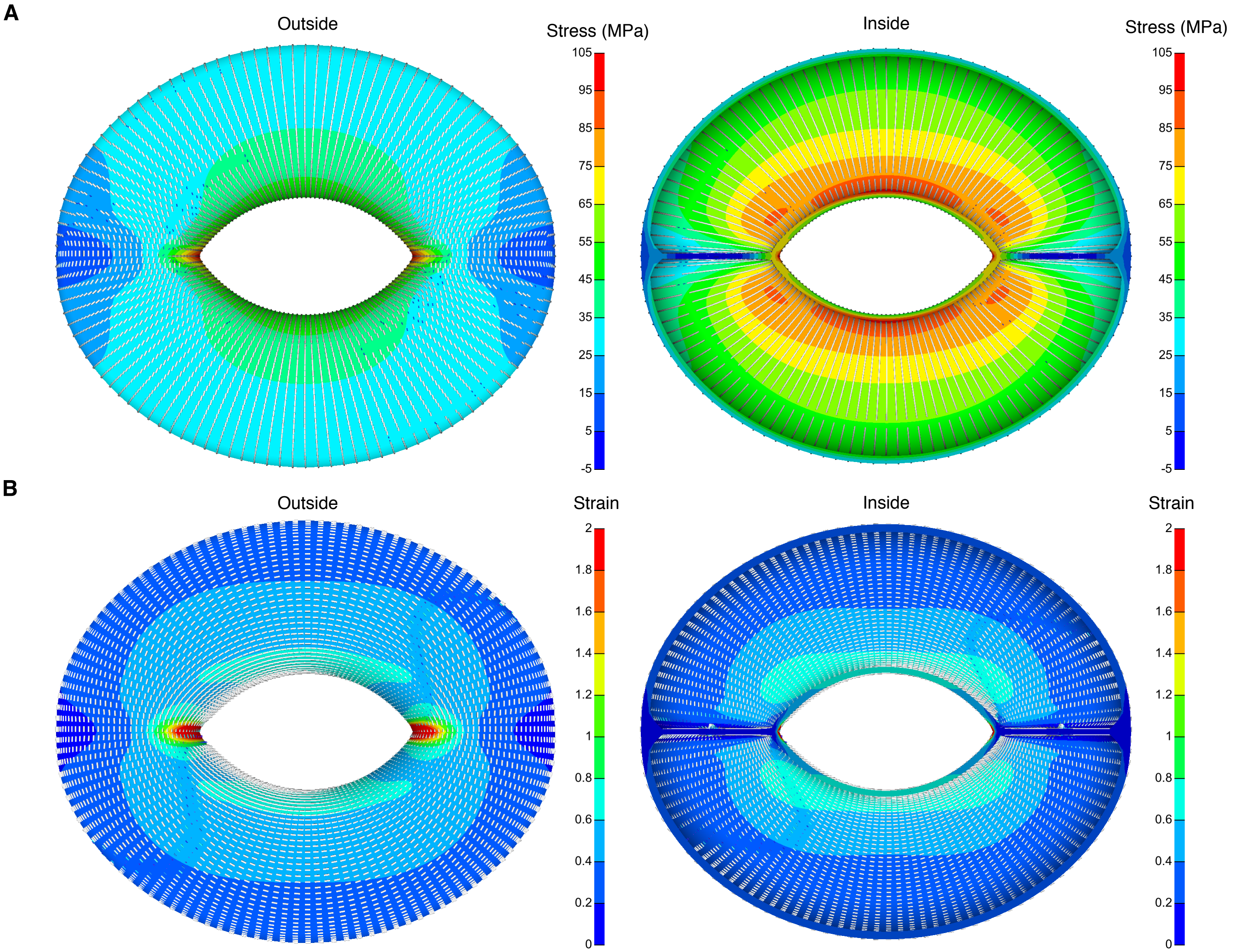

Figure S4. Stress and strain directions show hoop rigidity and longitudinal guard cell lengthening. **A**, Distribution of the first principal stress on the outside (left panel) and the inside (right panel) of the open stoma. **B**, Distribution of the first principal Lagrange strain on the outside (left panel) and the inside (right panel) of the stoma, with the strain limited to 2. The stomatal dimensions are given in Table 1 and the cell wall parameters are the 'Inferred #1' values in Table 2.

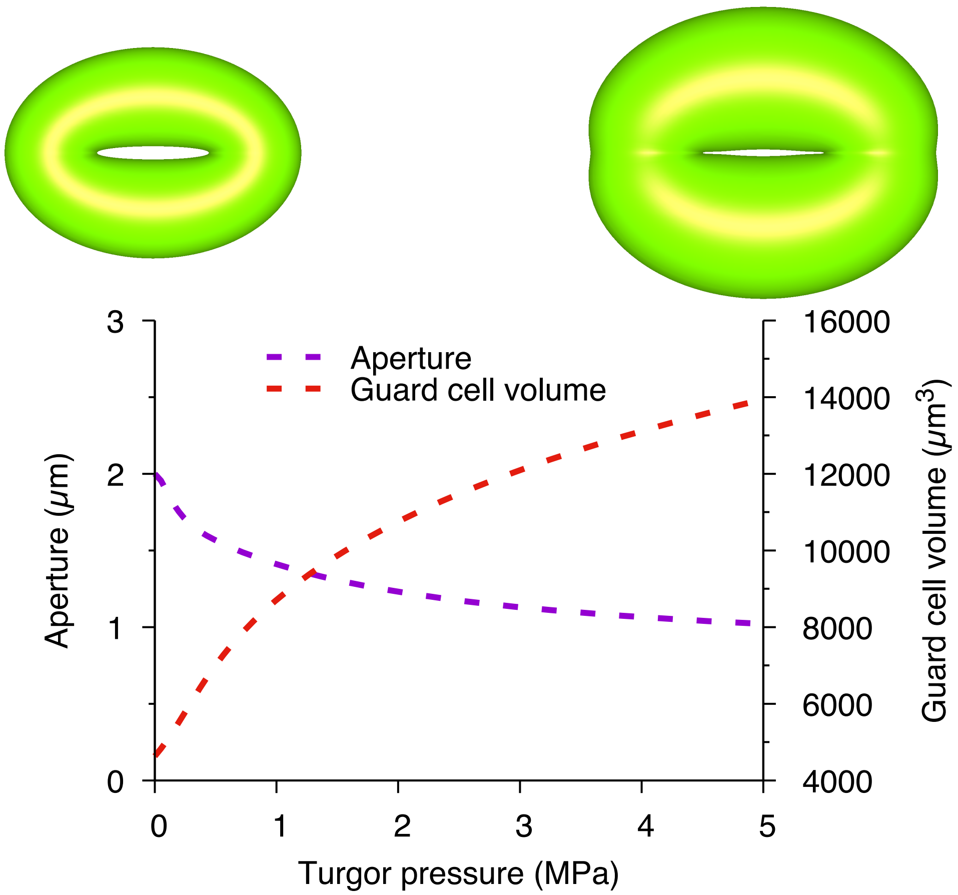

Figure S5. Circumferentially-oriented cellulose fibres are critical for proper stomatal function. The turgor pressure is increased from 0 to 5 MPa in a stoma with strain-stiffening, isotropic cell walls. The guard cells swell and stoma closes its pore. The stomatal dimensions are given in Table 1 and the cell wall matrix parameters are the same as the 'Inferred #2' values given in Table 3. Stomata images show the initial and final shapes, and are shaded to provide perspective.

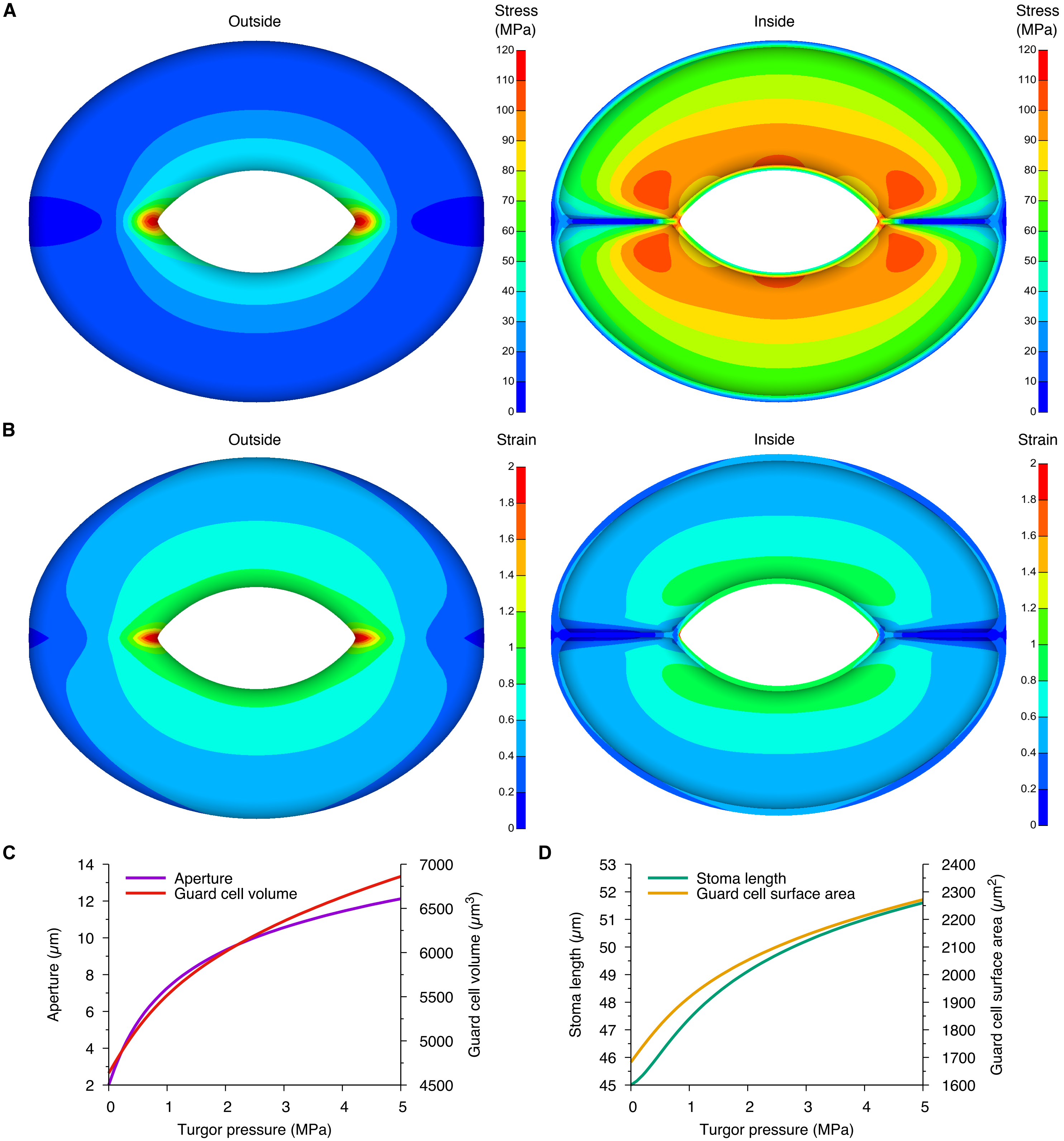

Figure S6. Stomatal opening induces stress and strain hotspots in guard cell walls. **A**, Distribution of the (effective) stress on the outside (left panel) and the inside (right panel) of the open stoma. **B**, Distribution of the (effective Lagrange) strain on the outside (left panel) and the inside (right panel) of the stoma, with the strain limited to 2. **C**, Increase in aperture (purple line) and guard cell volume (red line) as the turgor pressure increases from 0 to 5 MPa. **D**, Increase in the stoma length (green line) and the inner surface area of the guard cell (yellow line) as pressure increases. The stomatal dimensions are given in Table 1 and the cell wall parameters are the 'Inferred #2' values in Table 3.

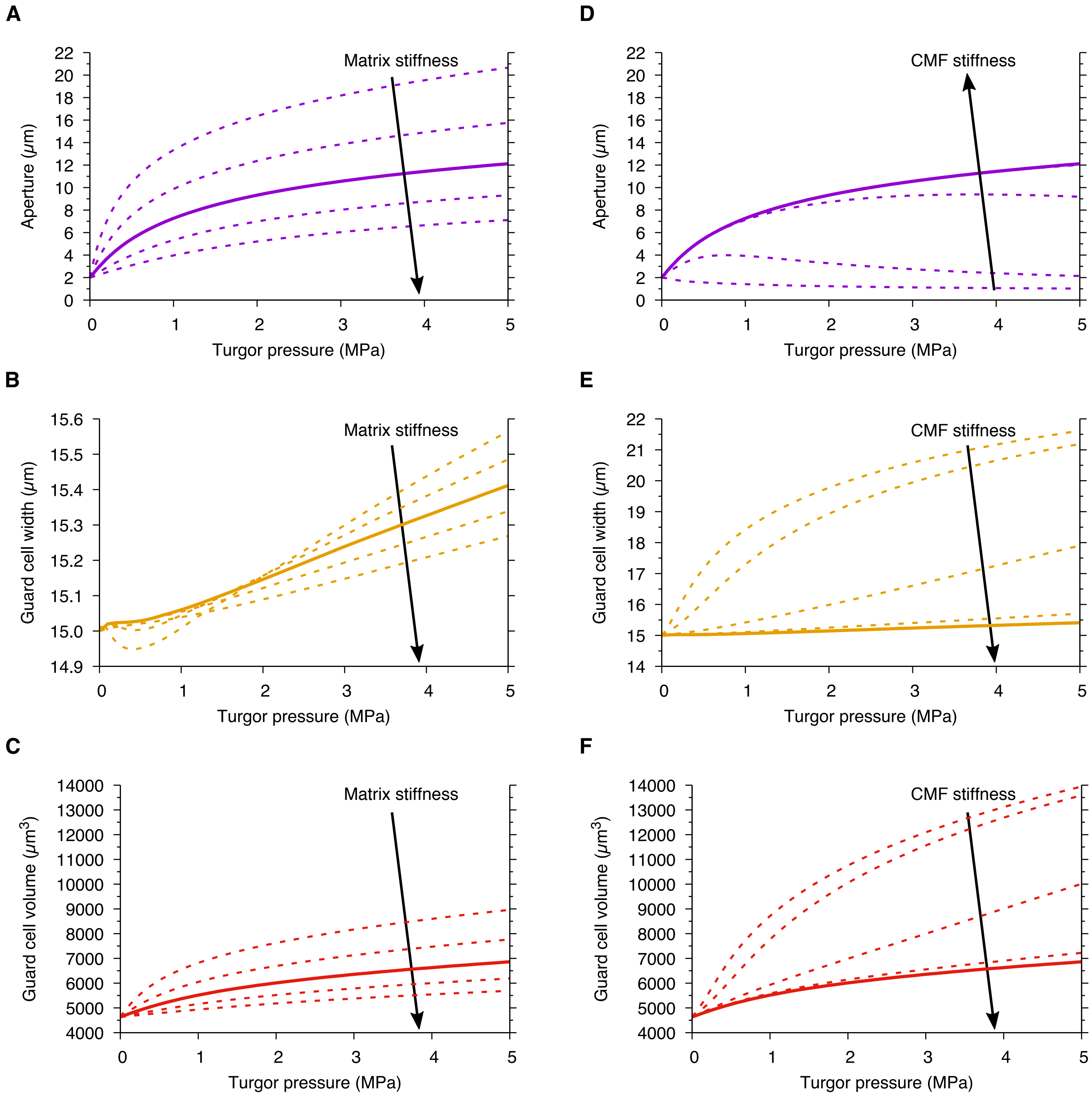

Figure S7. Aperture is oppositely affected by a stiffer cell wall matrix vs. stiffer CMFs. Sensitivity of the aperture (**A**, **D**), guard cell width (**B**, **E**) and the guard cell volume (**C**, **F**) to changes in the cell wall parameters. **A-C**, Sensitivity to a change in the initial shear modulus, G_0. The value of G_0 is 0.25, 0.5, 2 or 4$\times$ the value of the 'Inferred #2' set in Table 3 (dashed lines). **D-F**, Sensitivity to a change in the CMF modulus. The fibre modulus is 0, 0.01, 0.1 or 0.5$\times$ the fibre modulus value of the 'Inferred #2' set in Table 3 (dashed lines). The stomatal dimensions are given in Table 1. The solid lines correspond to the 'Inferred #2' cell wall parameter values in Table 3.
