# Supplementary material for: A computational approach for inferring the cell wall properties that govern guard cell dynamics
Source: Plant J. 2017 Aug 23;92(1):5–18. doi: 10.1111/tpj.13640 (PMC5637902; doi:10.1111/tpj.13640)
Supplement: Supplementary file 2 — Table S1. Change in stomata geometry of the cell wall genotypes of Arabidopsis as a function of the cell wall material property parameters. [file TPJ-92-5-s002.docx]

| **Change in matrix stiffness** | **Genotype** | **Pore width** | **Pore length** | **Stoma length** | **Guard cell width** |
| --- | --- | --- | --- | --- | --- |
|  | Col-0 | 1.97 (2.05 / 1.91) | 9.43 (9.45 / 9.41) | 30.53 (30.58 / 30.48) | 10.30 (10.31 / 10.28) |
|  | *irx8* | 2.20 (2.26 / 2.14) | 11.52 (11.53 / 11.51) | 30.74 (30.77 / 30.71) | 10.30 (10.31 / 10.29) |
|  | *pmr5* | 2.16 (2.25 / 2.09) | 10.71 (10.74 / 10.69) | 30.28 (30.34 / 30.23) | 10.43 (10.44 / 10.41) |
|  | *pmr6* | 2.52 (2.62 / 2.43) | 11.44 (11.48 / 11.42) | 29.17 (29.23 / 29.11) | 11.53 (11.54 / 11.51) |
|  |  |  |  |  |  |
| **Change in fibre stiffness** | **Genotype** | **Pore width** | **Pore length** | **Stoma length** | **Guard cell width** |
|  | Col-0 | 1.97 (1.91 / 2.03) | 9.43 (9.41 / 9.45) | 30.53 (30.51 / 30.54) | 10.30 (10.31 / 10.28) |
|  | *irx8* | 2.20 (2.15 / 2.24) | 11.52 (11.50 / 11.53) | 30.74 (30.72 / 30.75) | 10.30 (10.32 / 10.29) |
|  | *pmr5* | 2.16 (2.09 / 2.22) | 10.71 (10.69 / 10.74) | 30.28 (30.27 / 30.30) | 10.43 (10.45 / 10.41) |
|  | *pmr6* | 2.52 (2.43 / 2.59) | 11.44 (11.42 / 11.47) | 29.17 (29.15 / 29.18) | 11.53 (11.55 / 11.50) |

**Table S1.** Change in the stomata geometry of the cell wall genotypes of Arabidopsis as a function the cell wall material property parameters. The first set of values show how the inferred values for the pore width, pore length, stoma length and guard cell width change when the stiffness of the cell wall matrix, G_0_, is decreased by 10%, or increased by 10%. The second set of values show how the inferred values change when the fibre stiffness, C_5_, is decreased by 10%, or increased by 10%. In each trio of values, the first value is the inferred measurement, while the second and third values corresponds to a 10% decrease and a 10% increase in the relevant parameter. Stiffening the cell wall matrix causes a decrease in all four measurements, while increasing the fibre stiffness only causes the guard cell width to decrease.
